# Silicone Oil-Grafted Low-Hysteresis Water-Repellent Surfaces

**DOI:** 10.1021/acsami.2c20718

**Published:** 2023-02-15

**Authors:** Anam Abbas, Gary G. Wells, Glen McHale, Khellil Sefiane, Daniel Orejon

**Affiliations:** † Institute for Multiscale Thermofluids, School of Engineering, The University of Edinburgh, Edinburgh EH9 3FD, Scotland, U.K.; ‡ Department of Mechanical Engineering, University of Engineering and Technology, Lahore 39161, Pakistan; § International Institute for Carbon-Neutral Energy Research (WPI-I2CNER), Kyushu University, 744 Motooka, Nishi-ku, Fukuoka 819-0395, Japan

**Keywords:** hydrophobicity, silicone oil grafting, low contact angle hysteresis, small/no contact line pinning surfaces, wettability tuning

## Abstract

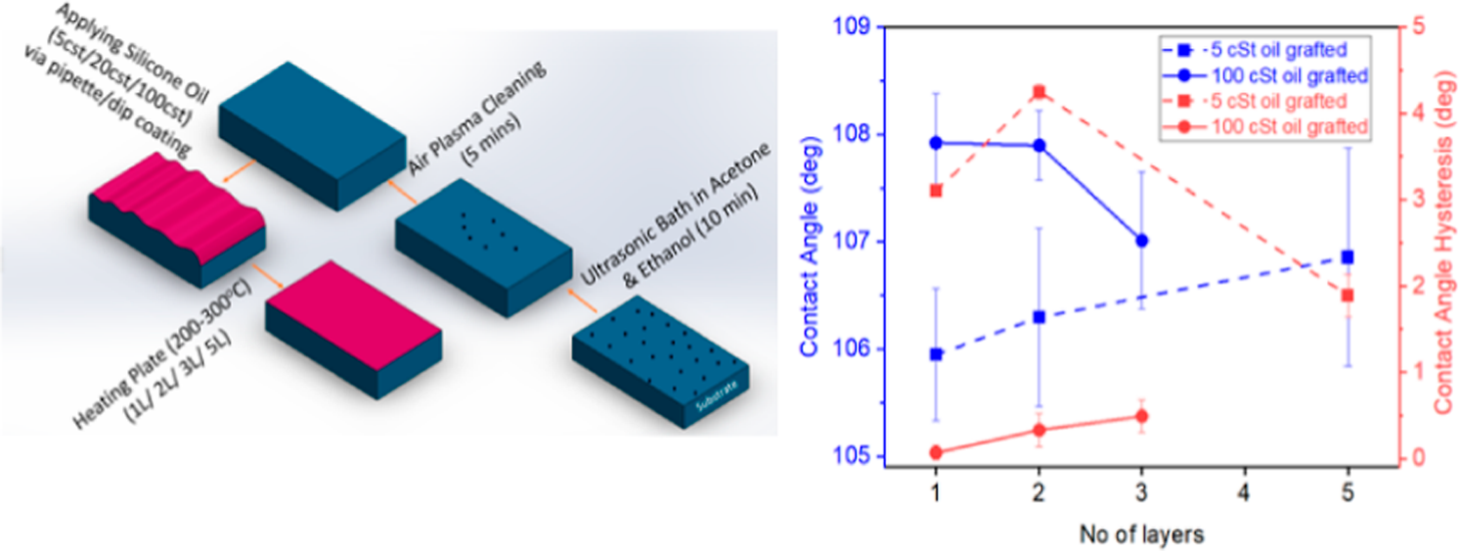

Wetting plays a major role in the close interactions between liquids and solid surfaces, which can be tailored by modifying the chemistry as well as the structures of the surfaces’ outermost layer. Several methodologies, such as chemical vapor deposition, physical vapor deposition, electroplating, and chemical reactions, among others, have been adopted for the alteration/modification of such interactions suitable for various applications. However, the fabrication of low-contact line-pinning hydrophobic surfaces via simple and easy methods remains an open challenge. In this work, we exploit one-step and multiple-step silicone oil (5–100 cSt) grafting on smooth silicon substrates (although the technique is suitable for other substrates), looking closely at the effect of viscosity as well as the volume and layers (one to five) of oil grafted as a function of the deposition method. Remarkably, the optimization of grafting of silicone oil fabrication results in non-wetting surfaces with extremely low contact angle hysteresis (CAH) below 1° and high contact angles (CAs) of ∼108° after a single grafting step, which is an order of magnitude smaller than the reported values of previous works on silicone oil-grafted surfaces. Moreover, the different droplet–surface interactions and pinning behavior can additionally be tailored to the specific application with CAH ranging from 1 to 20° and sliding angles between 1.5 and 60° (for droplet volumes of 3 μL), depending on the fabrication parameters adopted. In terms of roughness, all the samples (independent of the grafting parameters) showed small changes in the root-mean-square roughness below 20 nm. Lastly, stability analysis of the grafting method reported here under various conditions shows that the coating is quite stable under mechanical vibrations (bath ultrasonication) and in a chemical environment (ultrasonication in a bath of ethanol) but loses its low-pinning characteristics when exposed to saturated steam at *T* ∼ 99 °C. The findings presented here provide a basis for selecting the most appropriate and suitable method and parameters for silicone oil grafting aimed at low pinning and low hysteresis surfaces for specific applications.

## 1 Introduction

Wetting is a surface property used to describe the behavior and interactions between liquid droplets and/or films and solid or liquid-like solid surfaces. By manipulating a surface’s outermost layer, either physically or chemically, we can control the interaction between solids and liquids. This finds application in many engineering, medical, and industrial fields such as anti-icing,^1^ antifogging,^2^ self-cleaning,^3,4^ antibacterial,^5^ water harvesting,^6^ anticorrosion,^7,8^ heat transfer,^9−13^ and biomedical.^14^ A great deal of effort has been dedicated to creating surfaces that have a low affinity to water by developing and making use of easy, fast, and simple fabrication methods and procedures.^15,16^ These efforts aim to transform wetting surfaces into nonwetting surfaces or high hysteresis surfaces into low hysteresis ones by modifying the intrinsic wettability of the outermost layer as well as the surface morphology.^17−19^ Wettability is dependent on two types of intermolecular interactions: (i) between the molecules of the liquid and the surface or adhesive interactions/forces and (ii) between the liquid molecules themselves or cohesive interactions/forces. Experimentally, the solid–liquid interactions and wettability are usually described in terms of apparent contact angle (CA), which is the CA displayed near the contact line upon the gentle deposition of a droplet on a solid surface. In addition, the advancing as well as the receding CA [the difference between both gives the contact angle hysteresis (CAH)^20^] and the sliding angle (SA), additionally provide a metric for the characterization of the droplet/film-surface pinning. Therefore, sustained efforts have been dedicated to developing new coatings and novel procedures aiming to minimize contact line pinning, promote high mobility, and eventually achieve as low a CAH as possible.

Different techniques have been utilized to alter the surface physicochemical composition as well as its morphology to transform the intrinsic hydrophilicity of solid surfaces into hydrophobicity. These include chemical vapor deposition (CVD), physical vapor deposition (PVD), and spin coating, among others. CVD and PVD techniques have been widely applied to turn hydrophilic surfaces into hydrophobic ones with a small CAH by depositing a conformal hydrophobic silane-type promoter, initially in a gaseous or solid form, onto an otherwise hydrophilic surface. Silverio et al.^21^ used a CVD process to coat various substrates, including glass and silicon. This changed the CA from less than 20° to 60–75° after 30 min of CVD exposure time, hence enhancing the nonwetting nature of the surface. Mena et al.^22^ also applied CVD to coat sapphire substrates with porous gallium nitride. The surfaces showed a CA of 96° after a deposition time of 45 min.

Apart from achieving a low CAH, the stability of the coating and hence keeping uniform CA and CAH for longer periods of time is also crucial. Zhao et al.^24^ used CVD to deposit polydivinylbenzene thin films on silicon wafers, which initially showed a CAH of 20° with a rapid decay in the receding CA as time passed and hence a consequent rapid increase in the CAH. Thermal treatment was done to stabilize this decay in the receding CA, resulting in a stable CAH of 20–30° even after 2 months. Further efforts to create hydrophobic coatings were carried out by Paxson et al.^25^ using CVD yielding surfaces with CAH of 5°. These CVD-coated surfaces enabled condensation in a dropwise manner, with the consequent enhancement on the heat transfer performance. More recently, Ma et al.^26^ have made use of a lipid-inspired high adhesive interface by depositing perfluorodecyltrichlorosilane via molecular vapor deposition and a conformal fluorine polymer CF_x_ via plasma-enhanced CVD, showing high-condensation heat transfer performance and durability over a year. In addition to these, data-driven predictions and/or machine learning can also be applied for the optimization of parameters, such as CA.^27^ On the other hand, Lee et al.^23^ made use of PVD technique to enhance the hydrophobicity of glass enabling condensation occurrence in a dropwise fashion. Stearic acid films of various thicknesses were deposited on substrates, and it was observed that the advancing CA increased in a stepper fashion than the receding CA, with the evident increase in CAH as the thickness of the deposited films increased, until it reached a plateau at 20° for films thicker than 20 nm. They also reported that a smaller CAH can be achieved when the deposited coating is continuous and homogeneous rather than heterogeneous as expected. We can note that CVD has received most of the attention in the past decade, and it is the most commonly used technique due to its ability to deposit a monolayer of the coating with very accurate and controlled properties, as well as higher purity when compared to PVD, while PVD has mostly been applied as a protective coating against chemicals or corrosion.

Although the functionalization techniques described above provide a desirable outcome in terms of low wettability and low pinning, they are, however, rather complex and difficult to fabricate as per the need for an environmental chamber and vacuum for the deposition of high-quality coatings. In contrast, spin coating of hydrophobic films can be carried out without the need for a controlled environment or vacuum, although there is need for a further annealing process. The annealing process via sol–gel spin coating was exploited by Bougharouat et al.^28^ on hydrophilic glass substrates, which resulted in a CA of approximately 92°. Furthermore, Syafiq et al.^29^ developed a three-step technique for creating transparent hydrophobic coatings on a glass surface. Along with high transparency, the surfaces showed a CA of 103.9°. Although these coatings showed good results in terms of wettability, transparency, and stability, the preparation method involved several steps and chemicals, making it not suitable as an easy and fast procedure.

One more recent promising approach for the fabrication of low-contact line-pinning surfaces is the use of slippery omniphobic covalently attached liquid (SOCAL) surfaces.^17,30−32^ This method involves dip-coating a substrate in an isopropanol solution of dimethyldimethoxysilane and sulfuric acid, followed by drying and rinsing the substrate with water, isopropanol, and toluene. The resulting surfaces showed very low CAH of approximately 1° (i.e., low static friction) with lower droplet mobility (high dynamic friction) when compared to other functionalization techniques providing similar or even higher CAH.^33^

Another novel methodology explored for inducing the hydrophobicity of a solid surface is via oil grafting. This grafting process consists of the insertion of polydimethylsiloxane (PDMS) brushes by evaporating an oil on a surface via heating or using ultraviolet (UV) radiation.^5^ Note that grafting of silicone at ambient temperature and in the absence of UV radiation is also possible and has been recently reported.^34^ The types of oils/lubricants used in the process can range from fluorinated solvents and silicone oils to mineral oils and ionic liquids. Silicone oils and mineral oils are used most commonly because of their chemical compatibility with various substrates and wide viscosity ranges with no change in their chemical properties.^35^ In this regard, Eifert et al.^36^ and Chen et al.^37^ proposed a simpler technique to create hydrophobic surfaces with a further step of lubricant impregnation on several substrates. Silicone oil is applied to the surface and treated thermally. As a result, oil evaporates, leaving behind PDMS brushes that attach to the original surface, either glass, aluminum, silicon, copper, stainless steel, or brass, conferring a hydrophobic behavior and an intermediate CAH (20°). After further impregnation of the same silicone oil within the PDMS brushes, stable lubricant-infused surfaces (LISs) with a CAH of 2.4° were attained by Eifert et al.^36^

Despite the promising results obtained from these findings, no guidelines or attempts on the optimization of various parameters, such as the grafting temperature, type of oil, volume of oil grafted, oil viscosity, substrate underneath, and number of layers grafted/thickness of the coating, have been considered. The optimization of such parameters is very critical for the accurate control of the wettability and/or contact line pinning on these silicone oil-grafted hydrophobic surfaces.

Hence, here, we report a methodological and systemic approach providing suitable conditions for grafting silicone oil on solid surfaces, enabling hydrophobicity coupled with the flexible tuning of the CAH. This proposed method is simple, as it may only require one step, and easy, as it does not need vacuum conditions. The prepared surfaces adopting different fabrication parameters are then characterized in terms of wettability showing a high CA ∼ 108°, low-to-intermediate CAH between 1 and 20°, and low-to-high sliding/roll-off angles between 1.5 and 60° for 3 μL water droplets. By optimizing the fabrication parameters, extremely low CAH below 1° can be attained, as reported here, which is still lower than the values reported on LIS by Eifert et al.^36^ The durability and stability as well as the changes in roughness of the prepared surfaces have also been investigated and reported. In summary, this work provides a framework to select the most suitable method for altering surface wettability and/or tuning contact line pinning via silicone oil grafting using the pipette/dip-coating method and thermal grafting. Surfaces created in this manner could be further impregnated with oil to create slippery lubricant-infused porous surfaces (SLIPS) or LIS.

## 2 Experimental Section

### 2.1 Materials

Plain silicon wafers with a smooth finish on one side, purchased from Si-Mat (Silicon Materials, Germany), are used as the hydrophilic substrate. Silicon wafers are diced into 1 cm × 1 cm samples. Silicone oils varying in viscosities (5, 20, 100, and 1000 cSt) are purchased from Sigma-Aldrich. Pure acetone and ethanol organic solvents (Sigma-Aldrich) are used for cleaning the silicon wafers in an ultrasonic bath (Fisher Scientific UK Ltd, FB15047). On one hand, silicon wafers are adopted for the fabrication procedure because of their very smooth finish on one of the sides, which rules out any effects of structures or surface roughness on both the wettability characterization and the grating coating method employed. On the other hand, silicone oil is selected as the grafted lubricant owing to its nontoxicity, environmental friendliness, low cost, and ready availability, as reported by Eifert et al.^36^ In addition, an air plasma cleaner (Henniker Plasma UK, HPT-200, 60 W, 15 sccm, 5 min) is used to further clean the samples from any organic compound adsorbed onto the surface prior to grafting. A heating plate (Fisher Scientific UK Ltd.) is used for grafting the silicone oil.

### 2.2 Cleaning Procedure

**Figure 1:**
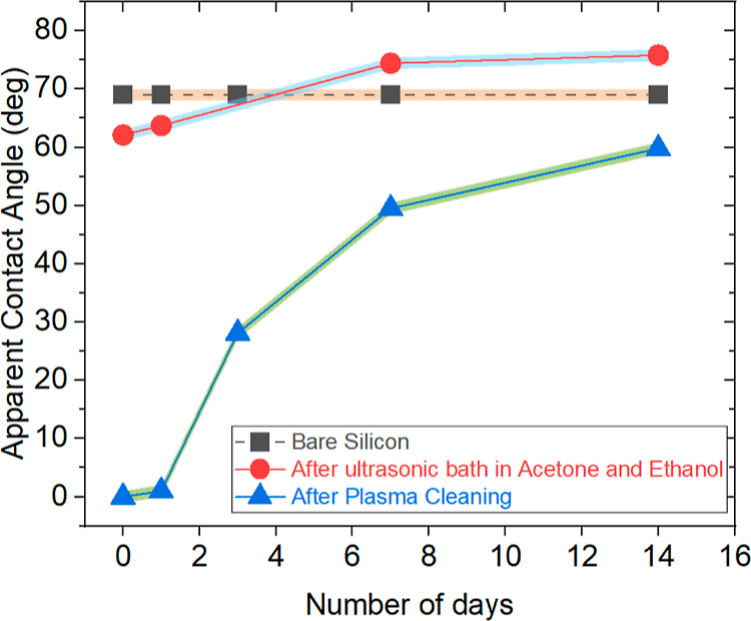
Apparent CA (deg) vs time (days) for a 3 μL water droplet on bare silicon as received, bare silicon cleaned with acetone and ethanol in an ultrasonic bath, and bare silicon cleaned with acetone and ethanol in an ultrasonic bath followed by air plasma cleaning. Note that the error bars are smaller than the represented symbols and hence the shaded area is used to represent the standard deviation of up to three measurements.

The first step before grafting is to clean the substrates. Diced silicon substrates are first cleaned in an ultrasonic bath of acetone and thereafter ethanol for 10 min, followed by rinsing with distilled water and drying with filtered compressed air. The samples are then further cleaned using the air plasma cleaner for 5 min to ensure that samples are free from any adsorbed component on the surface such as volatile organic compounds^38^ prior to the silicone oil grafting. The plasma-cleaning step is performed to ensure the complete spread of silicone oil on substrates since the presence of hydroxyl groups after plasma cleaning enhances surface wettability^39^ and provides better uniformity and stability of the oil grafted. Surfaces prepared without plasma cleaning still show good results in terms of wettability and low CAH, as shown in the Supporting Information Section SI-1. The CAs before and after solvent cleaning and after air plasma cleaning are measured at different laboratory ambient exposure times, which are presented in Figure 1. It is observed that a water droplet spreads completely on the sample immediately after air plasma cleaning and within 1 day from the plasma treatment.^38^ After that, finite CAs of 28°, 49°, and 59° are reported after 3, 7, and 14 days, respectively (Figure 1). Immediately after organic solvent cleaning, CAs are consistently above 60°, independent of the ambient exposure time (also shown in Figure 1). Hence, it can be anticipated that after air plasma cleaning, the samples are free of any adsorbed organic compound,^40^ and the silicone oil can then interact and bond directly to the surface.

### 2.3 Fabrication Procedure

**Figure 2:**
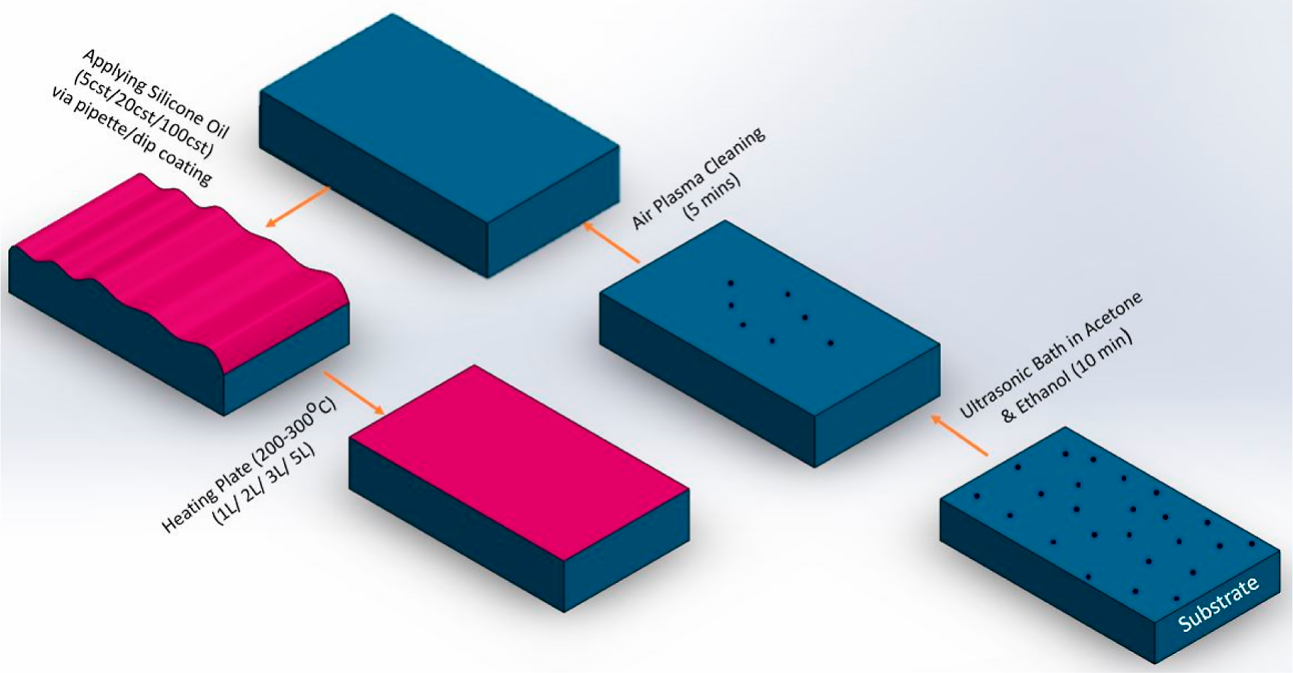
Schematic diagram of the experimental grafting fabrication procedure, including organic solvent cleaning, air plasma cleaning, application of the oil, and heating/grafting.

The fabrication process is depicted in the schematic diagram shown in Figure 2. A specified volume (3/5/10 μL) of silicone oil with different viscosities (5/20/100/1000 cSt) is applied evenly on the silicon substrate, after air plasma cleaning, using a pipette and/or via the dip-coating method; further details are provided below. After ensuring that the silicone oil has spread evenly over the surface, samples are then placed on a heating plate (200–300 °C) for sufficient time to allow all the oil to evaporate, leaving behind the surfaces with PDMS brushes grafted/attached to the surface.^5,37^ Multiple grafted layers (1/2/3/5 layers) are also investigated. The different grafting parameters investigated are summarized in Table 1.

**Table 1:**
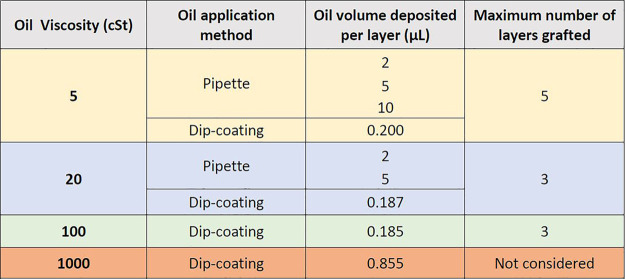
Summary of Grafting Parameters for Various Viscosity Oils

We note here the occurrence of nonuniform evaporation of silicone oil during grafting in the case of medium/high-viscosity oils (20, 100, and 1000 cSt), which have been applied evenly following the pipette method at grafting temperatures of 250–300 °C. Hence, to ensure the deposition of a uniform thin layer of high-viscosity oils, the dip-coating method, with withdrawal speeds of 0.05 mm/s for 20 cSt oil, 0.01 mm/s for 100 cSt oil, and 0.01 mm/s for 1000 cSt oil, is adopted along with low-grafting temperatures. The effect of grafting temperatures is briefly described in Supporting Information Section SI-2. These withdrawal speeds deposited approximately 0.187, 0.185, and 0.855 μL of oil for each coated layer, according to the Landau–Levich–Derjaguin (LLD) equation.^41^ We note here that the LLD equation estimates the thickness of the oil layer, which is then multiplied by the area of the substrate to estimate the approximate volumes reported above. Thereafter, silicone oil is then grafted on the surface, making use of the heating plate at 200 °C for 100 and 1000 cSt oils and at 250 °C for 20 cSt oil. A smooth surface finish is observed after the evaporation of 20 and 100 cSt oils; however, for 1000 cSt oil, nonuniform evaporation and nonuniform deposition of the oil on the surface were apparent. Hence, the present grafting method is deemed not suitable for such high-viscosity oil and hence oils with of viscosities higher than 100 cSt are not further considered within this investigation. 5 cSt oil grafted samples are also prepared via the dip-coating method (withdrawal speed: 0.2 mm/s, volume deposited: 0.2 μL^42^) to allow comparison between the two oil application methods for this low-viscosity oil.

### 2.4 Wettability, CAH, SA, and Surface Characterization

**Figure 3:**
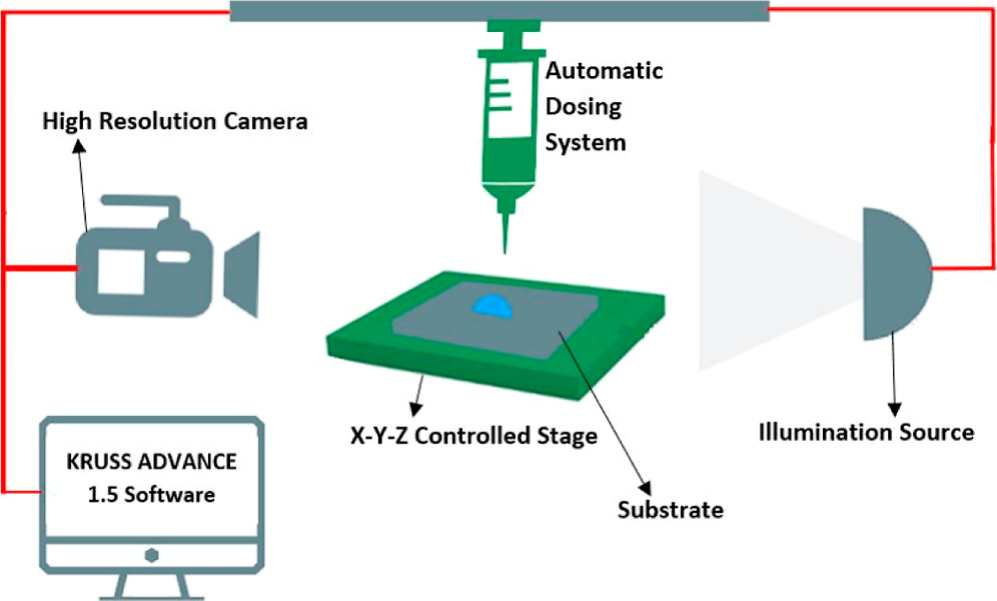
Schematic diagram of the DSA100 experimental setup, including the KRÜSS Advance software used to record videos for apparent CA, CAH, and SA characterization.

**Figure 4:**
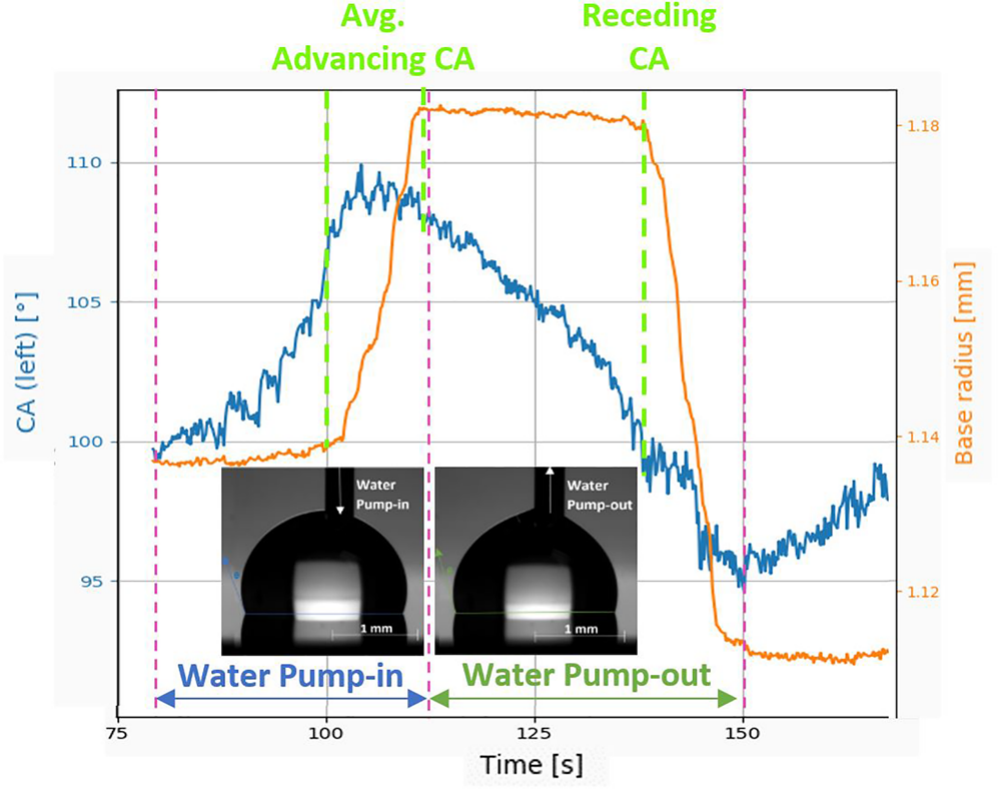
Experimental procedure for measurements of the advancing CA and receding CA using pyDSA.^44^ A water droplet of controlled initial volume is placed on the silicone oil grafted sample surface, and water volume is then added and withdrawn (indicated by white arrows in droplet snapshots) for measuring the advancing CA and the receding CA, respectively. Both the evolution of the CA (deg) and that of the base radius (mm) are then extracted and represented. Advancing CA is the average of CA values over a range when there is small variation in CA while the base radius is still increasing. Receding CA is the CA value when the base radius starts decreasing.

The fabricated surfaces are then characterized in terms of wettability or apparent CA, CAH, and SA. The working fluid used is deionized water with a surface tension of 72 mN/m at ambient temperature. A drop shape analyzer DSA100 from KRÜSS (KRÜSS Gmbh, Hamburg, Germany), comprising of an automated dosing system including a syringe and a needle, a CCD camera, a back light, and a manually controlled *x*–*y*–*z* stage, is used to deposit deionized water droplets of controlled volume of 3 ± 0.5 μL on different fabricated samples. The schematic of the setup is shown in Figure 3. Videos of the droplet during and after deposition are recorded using the DSA100 goniometer system, and these are analyzed using the pyDSA, an in-house droplet shape analyzer software.^43^ The apparent CA is measured after the gentle/slow deposition of a droplet on the surface. In order to retrieve the CAH, upon droplet deposition, water is added and withdrawn at the rate of 0.2 μL/s to obtain the advancing CA and receding CA, respectively. These are used to calculate the CAH = advancing CA – receding CA. The SA on the other hand is measured as the tilting angle at which a 3 μL water droplet rolls off from the surface. All experiments are performed at ambient room conditions, that is, at ambient temperature of 23 ± 3 °C, relative humidity of 38 ± 2%, and ambient pressure of 1 atm. Figure 4 shows the method followed to measure the advancing CA and receding CA using pyDSA.^44^ Here, the advancing CA is calculated as the average CA over a range of values at the initiation of the base radius motion, and the receding CA is obtained as the CA value when the base radius starts to decrease.

## 3 Results and Discussion

The prepared samples are characterized in terms of apparent CA, CAH, and SA. All experiments are repeated three times for SA and CAH, while for apparent CA, an average of the CA at four different sample locations (repeated three times at each location: center, edge, corner, and corner) is utilized. The results obtained are then presented and discussed in terms of different fabrication parameters as follows: in Section 3.1, the effect of oil viscosity; in Section 3.2, the number of layers; in Section 3.3, the volume of oil deposited; and in Section 3.4, the surface roughness. We note here that the grafting procedure applied renders the surfaces hydrophobic, that is, with CAs above 90° for all the fabrication parameters studied albeit with different values of CA, CAH, and SA, as will be discussed next. In addition, we subject certain samples to mechanical vibration, chemical as well as steam stability tests, which are introduced and discussed in Section 3.5.

### 3.1 Effect of Oil Viscosity

**Figure 5:**
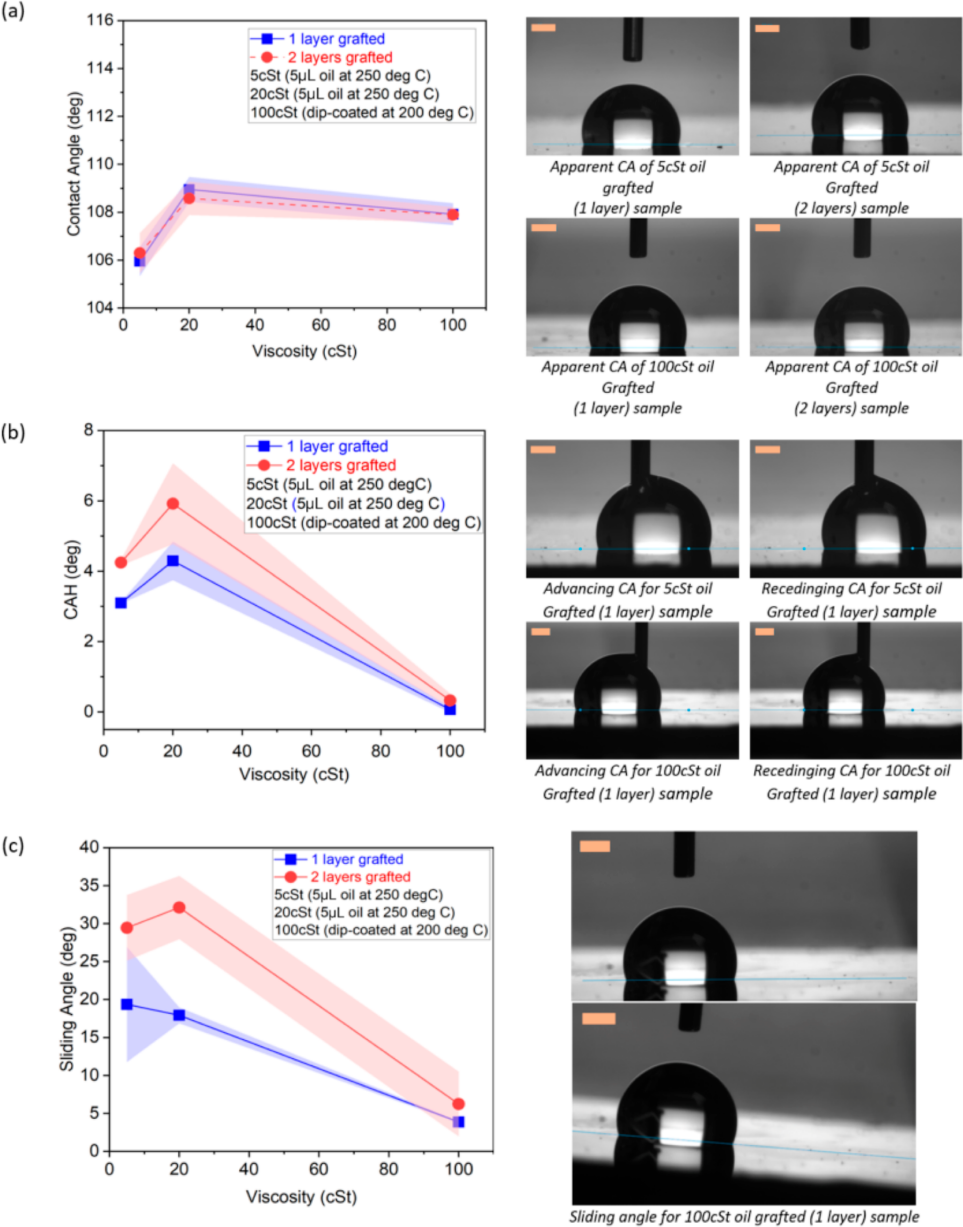
(left) Effect of viscosity and number of layers for a 3 μL water droplet on (a) apparent CA, (b) CAH, (c) SA, and (right) snapshots providing experimental observations of the droplets on different substrates for (a) wetting, (b) CAH as difference between advancing and receding CAs, and (c) SA. CAH and SA data for 5 and 20 cSt dip-coated samples are not represented, as they showed pinning even for a vertically tilted surface. The lines joining the points in graphs are not trend lines but merely connecting various data points without taking into account the standard deviation. The scale bar is 0.5 mm.

Various viscosity oils (5, 20, and 100 cSt) are utilized to address the effect of viscosity on the apparent CA, CAH, and SA, as all these parameters are related, to some extent, to surface wettability and contact line pinning. Table 1 provides a summary of the grafting parameters and the lubricant deposition method for various viscosity oils. On the one hand, the apparent CA displays values equal to or above 106° and increases approximately by 3 ± 1° as the viscosity of the grafted oil increases from 5 to 20 cSt, whereas no apparent change (just a slight decrease accounted for within the standard deviation) is noticed when comparing 20–100 cSt grafted oils (Figure 5a). On the other hand, when looking at the CAH and the SA represented in Figure 5b,c, respectively, an increase followed by an abrupt decrease of the CAH and the SA are reported when the silicone oil viscosity increases from 5 to 20 cSt and from 20 to 100 cSt, respectively. Results presented in Figure 5 for 5 cSt oil are for samples prepared via the pipette method, while those prepared via the dip-coating method show complete contact line pinning and high SA and CAH. In the case of 100 cSt oil grafted samples prepared via dip-coating, low SA and CAH are achieved along with an apparent uniform surface finish. 100 cSt oil grafted samples prepared via dip-coating empowered the lowest of the CAH and SA reported with values below 0.5° and 6°, respectively. This is a remarkable 20-fold decrease in CAH, when compared to earlier works^36^ reporting a CAH of 20°, and a 2-fold decrease in the SA when compared to Chen et al’s^37^ (SA of 10° for a 5 μL water droplet). The extremely low CAH reported here is analogous to that recently reported on SLIPS/(LISs)^36,45,46^ and/or SOCAL.^17,30,32^Figure 5a additionally includes CA experimental results for the same surfaces with two layers, which show no major differences upon the addition of a new layer, which is expected as the wettability of the surface is mainly governed by the physicochemical properties of the uppermost layer.^38^ While there is no obvious change in the apparent CA upon implementation of the second layer, an increase in both CAH and SA is observed for all oils, although the effect is more pronounced for 5 and 20 cSt oil viscosities than for higher viscosity ones. Rather similar values of CAH and SA are reported independently of the presence of one or two layers for 100 cSt prepared by dip-coating. The effect of number of layers is further addressed in Section 3.2.

### 3.2 Effect of Number of Layers

**Figure 6:**
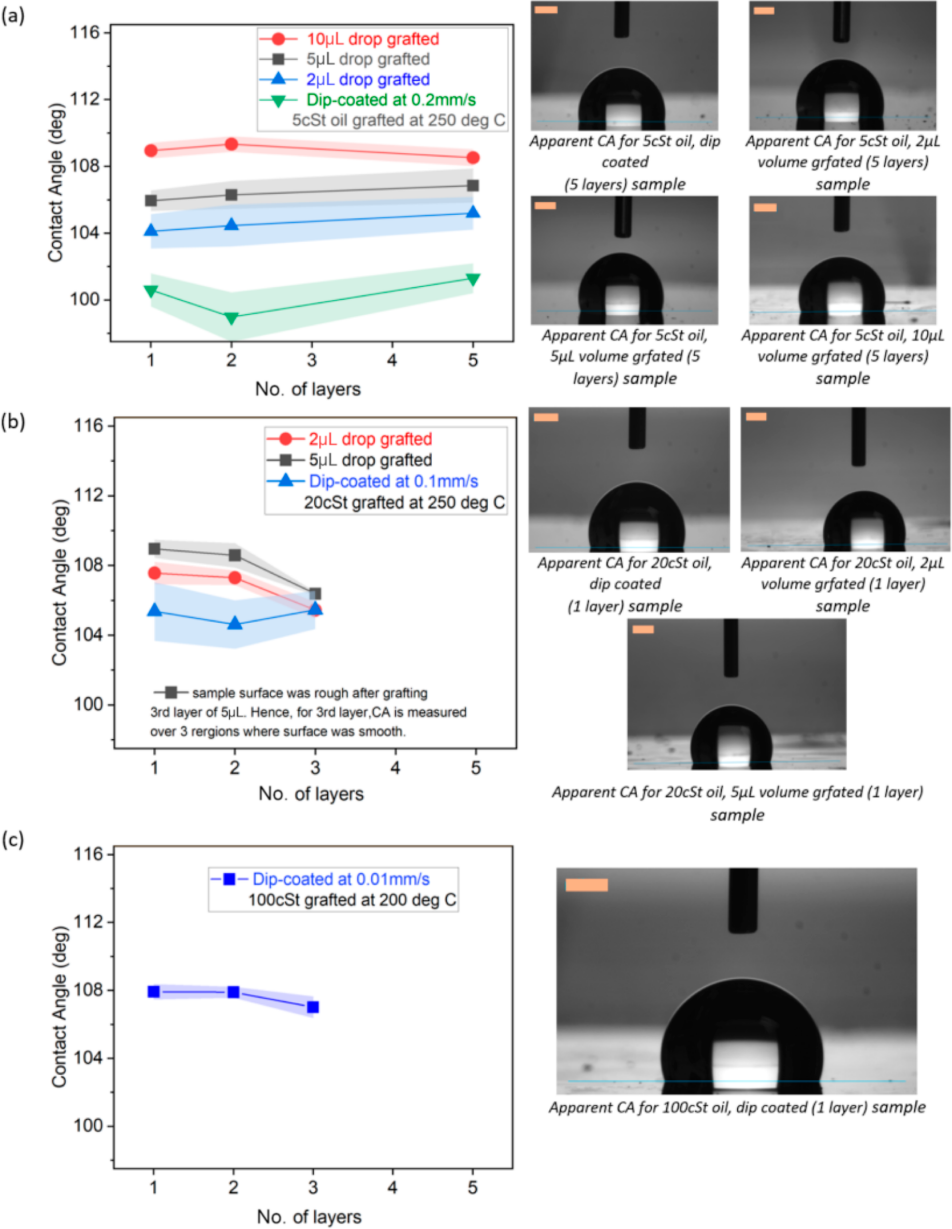
(left) Effect of the number of layers on apparent CA and (right) snapshots upon droplet deposition for a 3 μL water droplet for (a) 5 cSt oil grafted at 250 °C, (b) 20 cSt oil grafted at 250 °C, and (c) 100 cSt oil grafted at 200 °C. Note that there are only three layers grafted for 20 and 100 cSt oil, because there is no significant improvement in the apparent CA, SA, and CAH by increasing the number of layers for high-viscosity oils, despite the rather longer times required for grafting multiple layers. The lines joining the points in graphs are not trend lines but merely connecting various data points without taking into account the standard deviation. The scale bar is 0.5 mm.

In addition to the oil viscosity, different numbers of layers (from one to five) are grafted on substrates, and the apparent CA, CAH, and SA are then reported in Figure 6. When looking at 5 cSt grafted oil, independent of the deposition method adopted, that is pipette or dip-coating methods, or the volume of oil deposited, there is no apparent change in the CA when increasing the number of layers as represented in Figure 6a, where all CA values are within the standard deviation. As no noticeable change in CA is appreciated after grafting one and two layers is observed for 5 cSt oil samples, characterization for three and four layers were skipped and hence results for five layers are then shown. After the grafting of the first layer, subsequent layers attach to the already grafted ones, and no obvious change in the apparent CA is observed.^47^

Nonetheless, the different deposited volumes and the different oil deposition methods do yield differences in the apparent CA ranging from 99° to 110° about all in the cases of low- and medium-viscosity oils of 5 and 20 cSt (Figure 6). The range of CAs observed is somewhat related to the amount of PDMS brushes deposited on the surface after grafting. To this end, in the case of 5 cSt oil grafted samples, the smallest volumes of oil applied using the dip-coating method (see Table 1) showed the lowest CA, while the highest volumes of 10 μL applied via the pipette method yielded the highest CA. Again, no apparent differences in the CA are reported when comparing the number of layers. The same trend is observed for 20 cSt oil grafted samples where no major changes are reported in the apparent CA when comparing the different numbers of layers. However, a slight decrease in the apparent CA is observed in the case of 20 cSt fabricated via the pipette method for low volumes (2 and 5 μL of grafted oil), as represented in Figure 6b. For 100 cSt oil represented in Figure 6c, the increase in the number of layers also showed a negligible change in the apparent CA with a value of approximately 108° for all cases, which is quite close to the value reported by Chen et al. (105°).^37^

**Figure 7:**
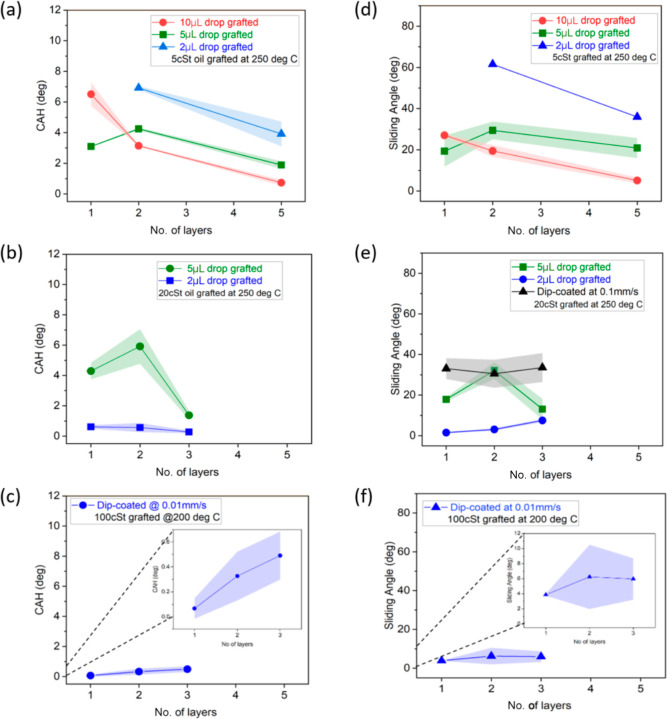
CAH variation with number of layers: (a) 5 cSt oil grafted at 250 °C, (c) 20 cSt oil grafted at 250 °C, and (e) 100 cSt oil grafted at 200 °C. SA variation with number of layers for 3 μL water droplet: (b) 5 cSt oil grafted at 250 °C, (d) 20 cSt oil grafted at 250 °C, and (e) 100 cSt oil grafted at 200 °C. Note that where there is no data on CAH or SA for dip-coated or 2 μL volume grafted samples (one, two, and five layers of 5 cSt and one, two, and three layers of 20 cSt), complete pinning of the contact line is observed; meanwhile, no further number of layers were investigated for 20 and 100 cSt silicone oils as three layers already provided excellent low CAH. The lines joining the points in graphs are not trend lines but merely connecting various data points without taking into account the standard deviation.

Although no major changes are observed when looking at the apparent CA, an overall decreasing trend in CAH and SA is observed as the number of layers increases for all samples with 5 cSt grafted oil (Figure 7a,d). A remarkable decrease is observed from a CAH of approximately 7° after the first layer down to 1° after the fifth layer for 5 cSt oil making use of 10 μL per layer via the pipette method. The SA also decreases in the order of tens of degrees after grafting 2 and 10 μL of 5 cSt oil via the pipette method after the fifth layer. For high CAH surfaces and prior to droplet movement, that is in the static regime, the coefficient of static friction between the droplet and the surface is also high, which leads to pinning of the droplet contact line and hence a high SA. For tilting angles above the SA, contact line pinning is then overcome by gravitational forces, and the droplet motion is initiated. The necessary force to keep the droplet in motion, that is in the dynamic regime, can be considerably lower than that needed for the onset of motion.^33,48^ The lower CAH and SA reported for multiple-layer grafted surfaces can be explained by the increased number and density of the embedded PDMS brushes, forming a denser chain structure, which effectively inhibits the direct interaction of water droplets with the underlying substrate,^47^ and hence droplets are more mobile. We shall stress here that in the case of low-viscosity silicone oil (5 cSt), the deposition of the oil via the dip-coating or pipette method, making use of small oil volumes before grafting, may not allow for the complete coverage of the substrate. This is supported by the high droplet pinning to the surface not being able to slide from the surface even for SA as high as 90°, in agreement with the SA on bare silicon substrates.

In the case of 20 cSt grafted oil making use of 5 μL oil per layer via the pipette method, a slight increase in CAH and in the SA are observed after the second layer, while a further decrease for a CAH below 2° and a SA near 10° are reported for the third layer, as shown in Figure 7b,e. The changes in the CAH and SA upon the increase in the number of layers are due to the fact that for medium-viscosity oils prepared via the pipette method (5 μL), nonuniform evaporation may occur during oil grafting, leaving behind a surface with a less uniform coating. For 5 and 20 cSt dip-coated samples, that is, the lowest of the oil volumes deposited before grafting, as included in Table 1, the contact line showed complete pinning even for a vertically tilted surface, presumably owing to the lack of complete grafted oil surface coverage.

Samples grafted with 100 cSt oil deposited via the dip-coating method yielded the lowest CAH among all samples after the first layer, with a slight upsurge in the CAH for further grafted layers. Nonetheless, CAH results for multiple layers were all below 1° and self-contained within the standard deviation, as seen in Figure 7c,f. These samples show the best performance in terms of low liquid–surface interactions because the longer molecular polymer chains contained in high-viscosity oils attach to the samples providing greater droplet mobility than in the case of shorter molecular polymer chains present in low-viscosity oils.^49,50^ In addition, the thickness of the grafted layer also increases proportionally with the viscosity of the oil (length of polymer chains) grafted^51^ and with the number of layers, thereby preventing any direct contact between the droplet and the silicon substrate, which results in high apparent CA as well as low CAH and SA.

### 3.3 Effect of Volume Deposited

The different volumes of oil grafted per layer are found in Table 1. In the case of 5 cSt silicone oil deposited on the substrate, via the pipette method, volumes are varied as 2, 5, and 10 μL, while 0.200 μL is deposited via the dip-coating method for each layer.^42^ In the case of 20 cSt silicone oil, 2 and 5 μL via the pipette method and 0.187 μL via the dip-coating method are grafted. While in the case of 100 cSt silicone oil 0.185 μL per layer are deposited. As shown in Figure 6a, the apparent CA increases from 101° ± 1° to 109° ± 1° as the volume of 5 cSt oil deposited increases from 0.2 to 10 μL, independent of the number of layers applied. Similarly, an increasing trend in the apparent CA is observed for 20 cSt oil grafted samples, which is also independent of the number of layers applied, as shown in Figure 6b. Independent of the method and the oil volume deposited, the apparent CA stays constant at a value of approximately 106° ± 1° for three or more layers. Conversely, higher volumes of oil deposited via the pipette method are not beneficial for high-viscosity 100 cSt oil and hence characterization and results are not provided within this paper. To sum up, independent of the oil deposition method and grafting parameters, a high volume of high-viscosity oil deposited via either the pipette or the dip-coating method induces nonuniform oil evaporation and oil displacement, with the consequent inhomogeneity of the coating deposition and the presence of pinning sites.

When looking closely at the SA for 5 cSt oil grafted samples, the SA typically decreases as the volume of oil applied via pipette increases (Figure 7d–f), except in the case of samples prepared via the dip-coated method and with 2 μL oil, which show contact line pinning of water droplets after one layer grafting even when the substrate is placed vertically with respect to the ground, that is at a tilting angle of 90°. An analogous behavior is observed for the CAH measurements for these samples, and as such, neither the CAH nor the SA is represented within Figure 7 for such low volumes of silicone oil grafting. However, when increasing the volume deposited via the pipette method, that is 10 μL of 5 cSt oil, the CAH reported is as low as 6.5°, which is 3-fold smaller than earlier values reported in the literature.^36^ We assume that the rather high contact line pinning reported in Ref 36 can be attributed to the small amount of oil deposited on the surface, to the rinsing step adopted after grafting, or to the lack of oxygen plasma treatement before grafting.

Next, when looking into 20 cSt grafted oil samples, the highest values of the SA are observed for the dip-coated samples (Figure 7e), unlike for 100 cSt grafted oil ones where dip-coating shows the lowest CAH and SA even after one single grafted layer. This is because in the case of low-viscosity and medium-viscosity oils, such as 5 and 20 cSt, the very small amount of oil deposited via the dip-coating method coupled with the high mobility of the PDMS brushes yields a nonhomogeneous surface and incomplete surface coverage of the silicon wafer underneath after evaporation, hence prompting droplet pinning.

To some extent, the volume of oil deposited/grafted on the surface is both proportional to the number of layers and to the amount of oil deposited for each step via the pipette method. Typically, greater volume and greater number of layers decrease contact line pinning and increase droplet mobility for low-viscosity oils (5 cSt). However, in the case of medium- and high-viscosity oils (20 and 100 cSt), the lowest of the volumes deposited via the pipette method and the low volume deposited via the dip-coating method yield the lowest CAH and SA, with these slightly increasing with the number of layers.

### 3.4 Roughness Analysis

**Figure 8:**
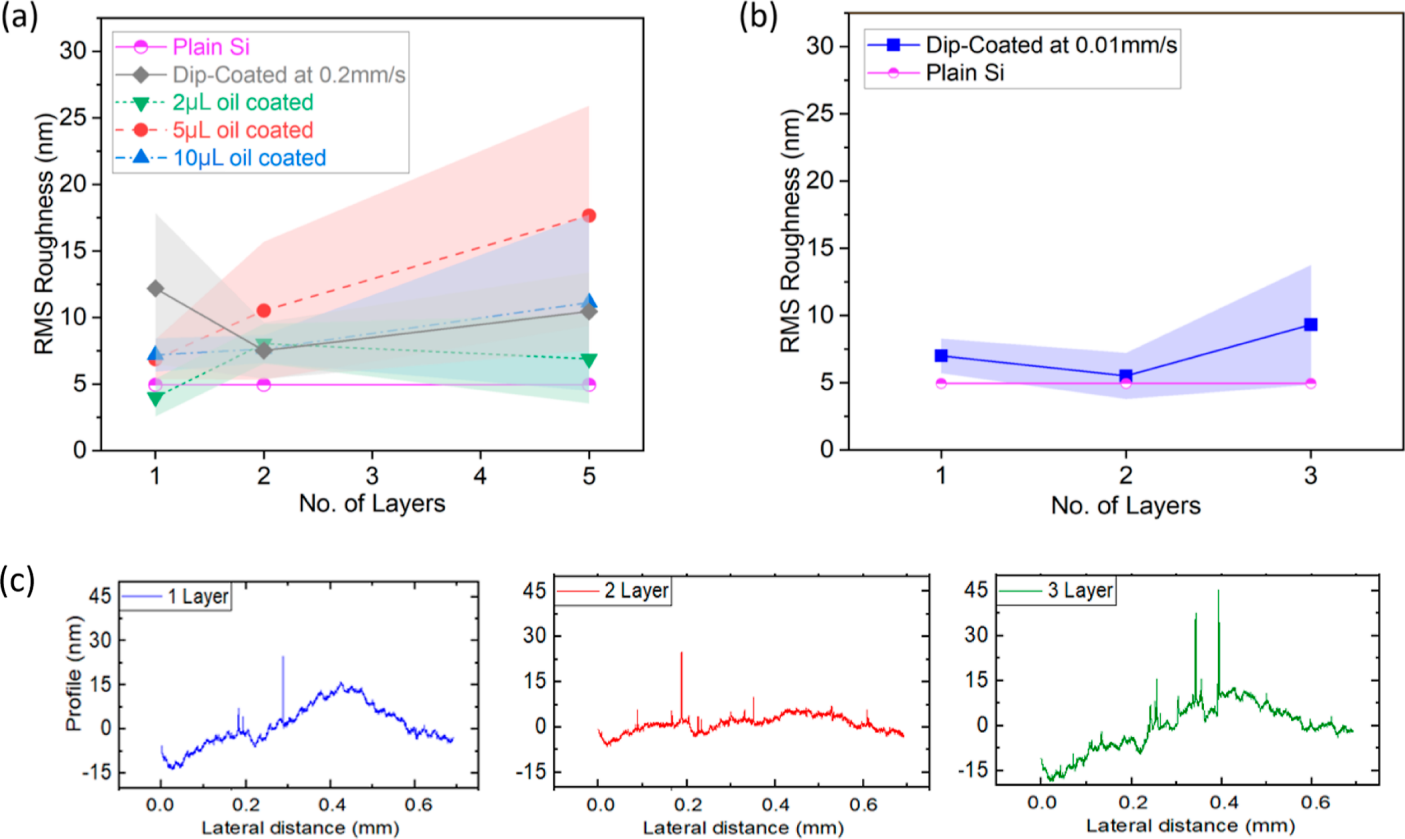
(a) RMS roughness of 5 cSt oil grafted samples, (b) RMS roughness of 100 cSt oil grafted samples, and (c) roughness profiles for different layers of 100 cSt oil grafted samples.

To understand the changes in surface morphology after oil grafting, the profile of the prepared samples is measured using a DektakXT Stylus Profiler with the Standard Manual X-Y Sample-Positioning Stage (Bruker, USA), and the root-mean-square (RMS) roughness values are calculated. It can be observed from Figure 8a,b that the average roughness values for all samples, independent of the oil viscosity, number of layers, volume of oil deposited, and deposition method, are within 20 nm, except for the cases where obvious heterogeneities on the surface are observed stemming from nonuniform oil evaporation. When looking at 5 cSt oil in Figure 8a and 100 cSt in Figure 8b, no clear increasing or decreasing trends in roughness are reported for the different volumes of oil deposited as well as for the different methods utilized and/or the number of layers applied. All values are within the same order of magnitude as the original silicone oil sample, and most of them are within the standard deviation, as shown in Figure 8a,b. The roughness profiles for one, two, and three layers of 100 cSt grafted oil samples are shown in Figure 8c. Hence, coated samples present very small roughness, which is comparable to that of plain silicone with no coating; however, the grafting method optimized here encompasses a remarkable increase in hydrophobicity and a decrease in contact line pinning, that is, SA as low as 10° and CAH below 1°.

### 3.5 Stability Tests

**Figure 9:**
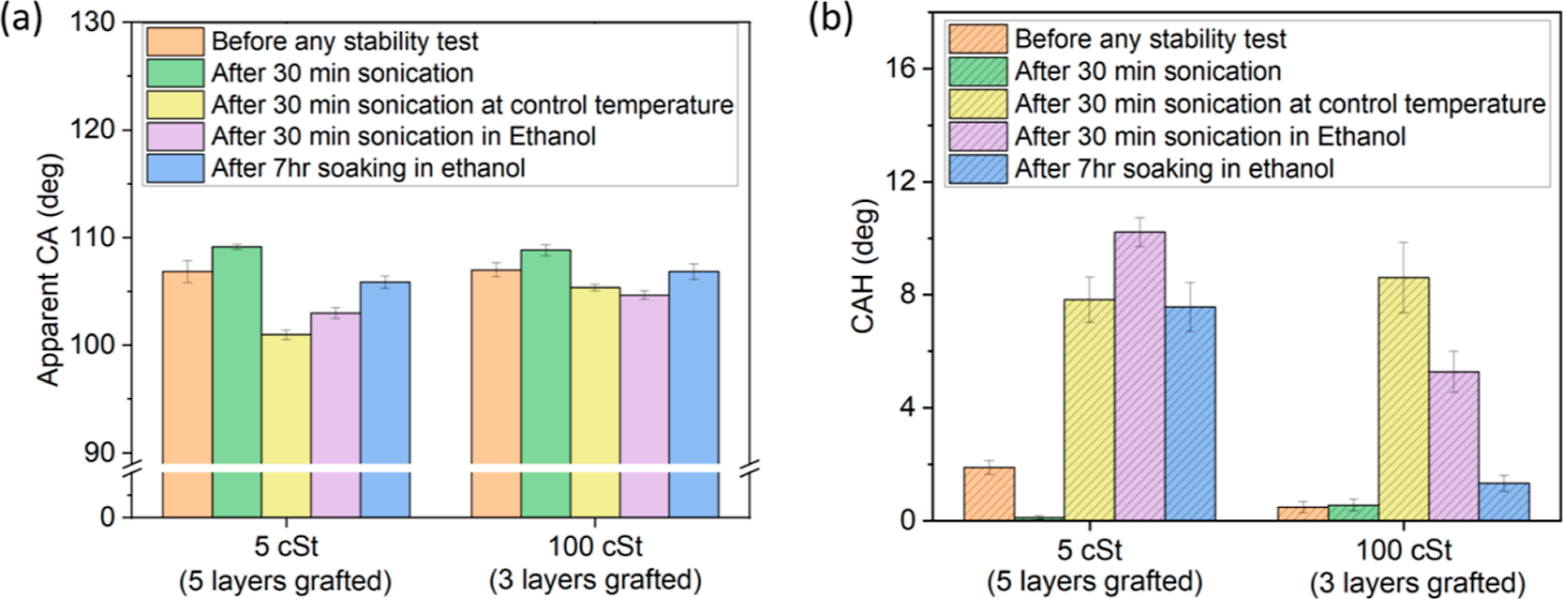
(a) Apparent CA and (b) CAH variation of 3 μL water droplet for various stability tests and grafted samples.

Five layers 5 cSt and three layers 100 cSt silicone oil grafted samples are tested under different conditions to provide preliminary insights on the stability of these coatings. The complete set of data and analysis of tests carried out for 5 and 100 cSt for the different number of layers can be found in the Supporting Information Section SI-3. To first check the mechanical stability of the coatings, the samples are placed in an ultrasonic bath for 30 min. Sonication means subjecting the samples to mechanical vibrations by placing them in a beaker in an ultrasonic bath of water. The temperature is maintained at 17–20 °C for sonication in controlled temperature tests. Samples are placed in a beaker containing ethanol, which is placed in an ultrasonic bath of water for sonication in ethanol tests. The error bars are attained by repeating each test three times. We note that as the ultrasonication proceeds, the temperature of the media and that of the samples increase to values near 50 °C due to the imposed mechanical vibrations. It is observed that in the case of increasing temperature, the stability worsens under mechanical vibration in ethanol tests (Figure SI4 and SI5 in the Supporting Information). Under solely mechanical vibrations with increasing medium temperature, the apparent CA slightly increases, while under mechanical vibrations at controlled temperature, the apparent CA decreases down to approximately 100° for both 5 cSt with five layers and 100 cSt with three layers. When looking into the results after mechanical vibration tests under noncontrolled temperature condition, the CAH remained below 1° showing lower or equal CAH than before the mechanical test. At controlled temperature, the CAH increases to 8° for both cases (Figure 9b). We note here that studying such an unexpected decrease in the CA and increase in the CAH when keeping the temperature of the mechanical testing near ambient is out of the scope of this work and may deserve further attention.

In parallel, stability tests via sonication in an ethanol pool are also carried out along with a further soaking test by immersing the samples in ethanol at room temperature for 7 h, which are represented in Figure 9 in shaded purple and shaded blue, respectively. An overall decrease in the apparent CA in the range of 3–5° is observed for all samples (both 5 cSt with five layers and 100 cSt with three layers), as indicated in Figure 9. This proves that the change in apparent CA is mainly due to the coupled mechanisms of sonication and temperature change, indicating the good strength of coatings in an organic solvent chemical environment at ambient temperature. When looking at the CAH, 100 cSt oil with three grafted layers showed better stability with CAH values below 6° and below 2° after mechanical vibrations in organic solvent and simple organic solvent soaking, respectively. In the case of 5 cSt with five layers of oil grafted samples, CAH above 8° are reported for these tests in an organic solvent environment. In terms of apparent CA, coatings are stable showing similar CAs after one single cycle/test, whereas in the case of the CAH, the increase in CAH is noticed for tests carried out in an organic solvent environment, which is more pronounced in the case of 5 cSt and five-layer samples, as represented in Figure 9a,b.

**Figure 10:**
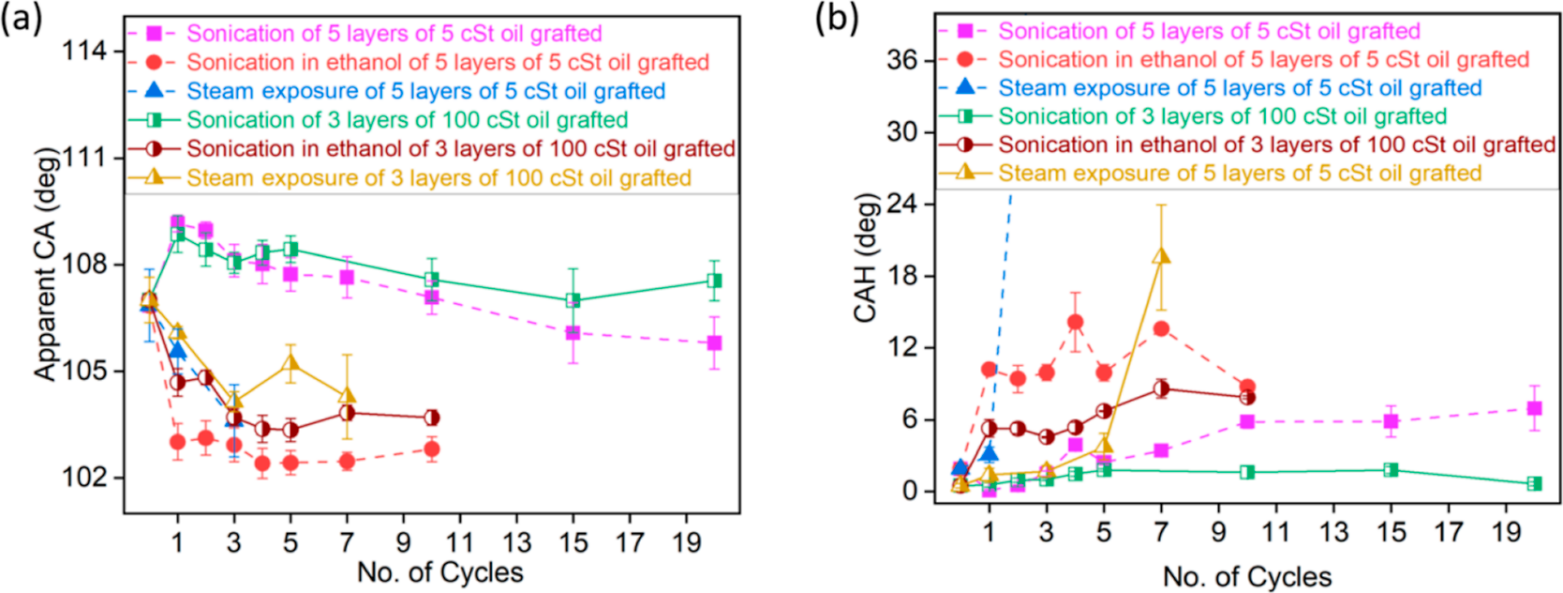
(a) Apparent CA and (b) CAH variation of a 3 μL water droplet under sonication, sonication in ethanol, and steam exposure tests. Each sonication and sonication in ethanol test durations are 30 min. Each steam exposure test duration is 5 min. The error bars are attained by repeating each test three times.

To further examine the stability of the coatings, tests are repeated for a total of 20 cycles under mechanical vibration or sonication and for a total of 10 cycles under mechanical vibration in ethanol, where each cycle lasts for 30 min, which are represented in Figure 10. The temperature is kept constant (17–20 °C) during sonication cycles. From Figure 10a, a small increase and then a decrease in the apparent CA are observed after two cycles of mechanical vibration, whereas in the case of mechanical vibration in ethanol, a decrease of a few degrees is observed after two cycles with 5 cSt and five layers being more pronounced than for 100 cSt and three layers. Mechanical vibration test results are comparable with results available in the literature, where no change in the apparent CA was observed after 20 min of sonication in ultrapure water and saline solution for LISs.^52^ When looking into the long-term effect on 5 cSt oil grafted with five layers under mechanical vibration, the apparent CA reached a plateau after 15 cycles, while a very small change is observed for 100 cSt oil grafted samples even after 20 cycles with an apparent CA of 108°. Under ultrasonication with ethanol, the apparent CA reached a plateau after the first cycle for both tested samples. After 10 cycles, the overall decrease in the apparent CA is approximately 4–7° for 5 cSt oil grafted with five layers and 3– 4° for 100 cSt oil grafted with three layer samples.

In terms of CAH, a decrease is observed within the first five mechanical vibration cycles for 5 cSt with five layers (Figure 10b) and thereafter an increase in the apparent CA follows. While for 100 cSt oil grafted samples, roughly no change in CAH is observed even after 20 cycles of mechanical vibration maintaining a CAH within 1 ± 1°. On the other hand, under mechanical vibrations in ethanol, an increase in the CAH is observed in all cases after the first cycle. After 10 cycles, an average CAH of 10° is reported for 5cSt and five layers, while an average CAH of 6° can be established for 100 cSt and three layers, which is still at least 3-fold smaller CAH than that reported by Eifert et al.^36^

To further investigate the stability of the PDMS brushes grafted in a humid and high-temperature environment, samples are subjected to high-temperature saturated steam (99 ± 1 °C) for different number of cycles with a duration of 5 min per cycle. For low-viscosity grafted oil, that is 5 cSt, the apparent CA decreases marginally after one cycle, but a considerable decrease was observed after three cycles with an apparent CA below 100° (Supporting Information, Section 2). Whereas, for high-viscosity oil grafted samples, that is 100 cSt, a 3–4° decrease in the apparent CA is observed after the first cycle, and no further change is reported even after seven cycles of steam exposure with an apparent CA above 104°. In terms of CAH, for 5 cSt oil grafted samples, a small increase is perceived after one cycle, while a substantial increase is observed afterwards. While for 100 cSt oil grafted samples, the increase in CAH is very small below 10° for two- and three-layer grafted samples even after five cycles of steam exposure. Thereafter, the CAH increases sharply with a value equal to or above 20°. This is coherent with the results reported by Liu et al.,^53^ where CAH below 20° is reported on PDMS brushes even after 40 h of exposure to the steam; however, in their case, the steam temperature was kept at 70 °C, while our saturated steam was at 99 °C. When comparing these two works, as the temperature of the steam increases (from 70 to 99 °C), the stability of PDMS brushes decreases. Overall, the 100 cSt oil grafted samples showed very good results in terms of wettability (high apparent CA), contact line pinning (low SA as well as low CAH), and stability, with low change in apparent CA, CAH, and SA, throughout the different tests. Although PDMS brushes can actually be utilized effectively for steam condensation,^54^ it did not work in our case. We note here that more research work focusing on the physical and chemical interactions between the grafted oil and the solid surface are needed for the development of more stable grafting procedures in harsh environments.

### 3.6 Analysis and Discussion

To summarize, we are able to achieve hydrophobicity and considerably low CAH values via our optimized procedure. In the case of low-viscosity oils, high volumes of oil via the pipette method for the oil deposition and a greater number of layers are preferred to ensure complete surface coverage, while for higher-viscosity oils, lower volumes making use of the dip-coating method to ensure uniform coverage of the surface are preferred so as to avoid nonuniform evaporation. To highlight, all oils can achieve CAH near few degrees after just one single step, though a specific oil deposition method and the right oil volumes are required. Among all samples, 100 cSt oil-grafted samples achieved via the dip-coating method showed minimum SA and CAH with values as low as <1° and <6°, respectively, after the first grafted layer, which remained almost constant even after grafting three layers (Figure 7c,f). Overall, a clear decrease in the SA as well as in the CAH is evident as the volume of oil applied increases, except for the high-viscosity 100 cSt silicone oil. Next, Table 2 provides characterization results on earlier reported works in the literature on grafted samples including the coating procedure as well as CA, CAH, SA, and roughness values, along with the comparison withthe present work. When looking into the apparent CA, all the different works included in Table 2 provide a similar value within the hydrophobic regime; however, both the CAH and SA differ depending on the fabrication procedure adopted. CAH ranges from <0.5° to 20°, while SA varies from <4° to 10°, with the lowest values reported for the present work under one-step dip-coating 100 cSt silicone oil grafting.

**Table 2:** Comparison of Previous Results from the Literature with the Present Work

author	coating	apparent CA (deg)	CAH (deg)	SA (droplet volume)	roughness (nm)	refs
Eifert et al.	grafting of silicone oil (5 cSt) on a silicon substrate via heating		20			(36)
Liu et al.	silicon substrate immersed in toluene solution containing DCDMS (to create PDMS brushes on the surface)	106	<4	4–7° (5–20 μL)	0.1	(53)
Tesler et al.	silicone oil (500 cSt) grafted on a silicon substrate via UV irradiation	106	10			(5)
Chen et al.	silicone oil (100 cSt) grafted onto a glass substrate via heating	105		10° (5 μL)		(37)
Abbas et al.	silicone oil (100 cSt) grafted on a silicon substrate via dip-coating and heating	108	<0.5	<4° (3 μL)	<20	present work

**Figure 11:**
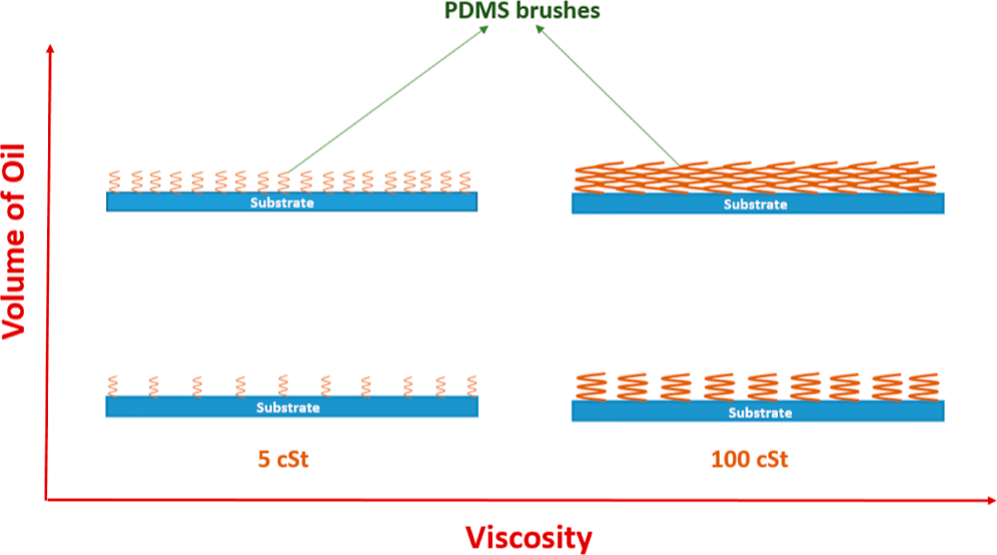
Schematic illustration of formation of PDMS brushes for low/high volumes and viscosities of silicone oils.

The optimized parameters for silicone oil grafting are summarized in Table 3 while Figure 11 illustrates the schematics on PDMS brushes grafting via low/high volumes of silicone oils with varying viscosities, where longer and denser polymer chains are attached on the surface in the case of high-viscosity oils.^49,50^ For low-viscosity oils, grafting low volumes results in some regions with absence of PDMS brushes or coating coverage causing the droplet to pin at different sites, which is undesirable; hence, high volumes and a high number of layers following the pipette method are preferred. However, in the case of high-viscosity oils, low volumes deposited via the dip-coating method are preferred, while the low number of layers minimizes the number of steps and fabrication time.

**Table 3:** Summary of Grafting Parameters for Various Viscosity Oils^a^

	low-viscosity oil		high-viscosity oil	
oil application method	pipette method*	dip-coating	pipette method	dip-coating*
volume of oil grafted	high*	low	high	low*
number of layers grafted	high*	low	high	low*
roughness after grafting oil	high	low*	high	low*

a * shows appropriate conditions for grafting silicone oil.

A recent review by Chen et al. provides a clear account on the effect of molecular weight or chain length of the grafted brushes on the grafting density and on CAH. On one hand, an increase in the molecular weight or chain length of the grafted PDMS brushes results in a decrease of the grafting density. On the other hand, a decrease and then an increase in the CAH is reported as the molecular weight or chain length of the PDMS brushes increases.^55^ In the present case, we observe a decrease in the CAH when increasing the silicone oil viscosity between 5 and 100 cSt. This is in agreement with the literature^55^ as in the case of 100 cSt oil the molecular weight or chain length is greater than for 5 cSt oil, so when grafted on substrate they create thick films of PDMS brushes with medium grafting density. This minimizes the direct contact of water droplet with the base substrate, which in turn reduces the pinning sites and eventually exhibits a small CAH. Further increase of the viscosity of the silicone oil may result in a lower grafting density, and as reviewed by Chen et al. a presumable increase in the CAH.^55^ In the case of low-viscosity oils, the shorter polymer chains attached to the substrate enhance the hydrophobicity of the surface. However, the thickness of the grafted chains is rather small in this case, which eventually exposes the substrate to the deposited droplets, that is pinning sites, which give rise to high CAH.^56^ In addition, Liu et al. also reported that the higher the thickness of the PDMS brushes, the lower the adhesion to water droplets. Hence, a thick and dense layer of PDMS brushes is deposited on the base substrate when medium-viscosity oils (100 cSt) are grafted, which ultimately enhances the mobility of the three-phase contact line on such surfaces as in the present case.^57^ This is also supported by the fact that the bigger island features on grafted samples (longer molecular chain lengths in our case) ensure the full coverage of the substrate, leaving no gaps between the grafted structures (PDMS brushes), which reduce the presence of pinning sites, and as such, a low CAH is observed on such surfaces.^55^

Besides silicon as the base substrate, additional experiments making use of polished copper substrates showed, on the one hand, similar apparent CA in the hydrophobic regime. On the other hand, CAH in the case of 5 cSt grafted oil increased considerably by 1 order of magnitude, whereas in the case of 100 cSt grafted oil, only an increase of 1 or 2° was observed when compared to silicon based ones. The presumably lower wetting and higher roughness of the copper substrates (as compared to the atomically smooth silicon wafer) would slow down or hinder the motion of the silicone oil contact line during grafting, which may result in less uniform and less homogeneous film deposition during grafting with the consequent droplet pinning enhancement. In addition, the different affinity of the PDMS brush attachment (especially the short chain length when grafting a low-viscosity oil) with the base substrate also may play a role in the motion of the water droplet contact line when characterizing the CAH. Further details on these results are provided in Section SI-3 of the Supporting Information.

Although it is not the primary focus of this communication, the stability of the grafted layers is of paramount importance for long-term applications. Nonetheless, to provide some preliminary insights on the stability of the different grafted surfaces; mechanical vibrations, chemical immersion, coupled chemical and mechanical vibrations, and steam environment, have been carried out and are included and discussed in Section 3.5, whereas further specific details of these tests and results are provided in Section SI-4 of the Supporting Information. Overall, 100 cSt oil-grafted samples show the highest stability even after 10 h of mechanical vibrations and after 5 h of sonication in an organic solvent (ethanol), which render these surfaces promising candidates for fluid manipulation. Findings reported here not only benefit the industry but also biomedical, microfluidics, engineering, and electronic applications.^1,2,4−6,8,13,14,58^

## 4 Conclusions

We have presented a systematic approach to optimize the fabrication parameters for preparing easy, one-step, low-CAH water-repellent surfaces by silicone oil grafting over bare silicon wafers. Silicone oils with viscosities ranging from 5 to 100 cSt have been applied using either a pipette or dip-coating method for mono- and multilayer/step deposition prior to grafting. During grafting, the oil evaporates, leaving behind the so-called PDMS brushes, rendering the surface hydrophobic with nonwetting CAs between 105 and 108° and a different range of SAs and CAH as a function of the fabrication procedure adopted. Optimized grafting parameters enabled a remarkable CAH below 1° for 100 cSt silicone oil, owing to the longer molecular chains of the PDMS brushes that are covalently attached to the surface. This extremely low CAH is comparable to current LISs and slippery, omniphobic, covalently attached liquid-like surfaces and an order of magnitude lower than that reported by earlier works using a similar grafting procedure. In addition, the parameters studied here offer the additional capability of tuning the CAH between 1 and 20° and SAs between 1.5 and 60° (for 3 μL water droplets), depending on the fabrication procedure. When looking at the different parameters studied, higher volumes of the deposited oil are preferred for low-viscosity oils, whereas low oil volumes as well as lower grafting temperatures are more appropriate for high-viscosity oils. Moreover, the prepared surfaces demonstrate good stability under various tests, with the 100 cSt oil-grafted sample showing the highest stability in terms of wettability, with CAs in the range of 103–107° and CAH below 9° even after 10 cycles of ultrasonication in ethanol. Our findings support the parameter optimization of an easy, fast, and universal approach to create hydrophobic low contact line pinning surfaces, which may be utilized to fabricate further LISs and/or SLIPSs where the stability of the coating, prior to impregnating a lubricant oil, is crucial for specific engineering and/or industrial applications.

## Supporting Information Available

Effect of plasma cleaning on wettability, effect of temperature, wettability analysis on a copper substrate, and detailed information on stability tests (PDF)
