# Supplementary material for: Silicone Oil-Grafted Low-Hysteresis Water-Repellent Surfaces
Source: ACS Appl Mater Interfaces. 2023 Feb 15;15(8):11281–95. doi: 10.1021/acsami.2c20718 (PMC9982814; doi:10.1021/acsami.2c20718)
Supplement: Supplementary file 1 — am2c20718_si_001.pdf [file am2c20718_si_001.pdf]

Supporting Information

Silicone Oil Grafted Low Hysteresis Water Repellent Surfaces

Anam Abbas[1,2], Gary G. Wells[1], Glen McHale[1], Khellil Sefiane[1], Daniel Orejon[1,3*]

[1] Institute for Multiscale Thermofluids, School of Engineering, The University of Edinburgh, Edinburgh EH9 3FD, Scotland, UK

[2] Department of Mechanical Engineering, University of Engineering and Technology, Lahore, Pakistan

[3] International Institute for Carbon-Neutral Energy Research (WPI-I2CNER), Kyushu University, 744 Motooka, Nishi-ku, Fukuoka 819-0395, Japan

Corresponding author: Dr. Daniel Orejon (d.orejon@ed.ac.uk)

SI-1. Effect of plasma cleaning on wettability

To understand the necessity of plasma cleaning step in fabrication process, some samples are prepared following the same procedure as detailed in section 2.3. of main manuscript but with exclusion of plasma cleaning step. These samples prepared without plasma cleaning are 1 and 5 layers of 5 cSt oil grafted as well as 1 layer of 100 cSt oil grafted. The resulting CA and CAH are shown Figure SI-1. There is negligible change in CA for five layers of 5 cSt oil grafted and one layer of 100 cSt oil grafted (Figure SI-1a). But 5cSt one layer grafted sample showed a decrease of ~8° in CA and increase of 25° in CAH. This is because of the incomplete coverage of oil on the substrate which yielded to pinning sites. Therefore when more layers are grafted on the top of first layer, the CAH decreased. One layer 100 cSt oil showed increase of ~5° in CAH, when prepared without plasma cleaning step (Figure SI-1b). Hence even without plasma cleaning, grafting more layers of low viscosity oil or grafting high viscosity oil still yields to high CA and low CAH (below 20° for low viscosity, below 5° for high viscosity oil).

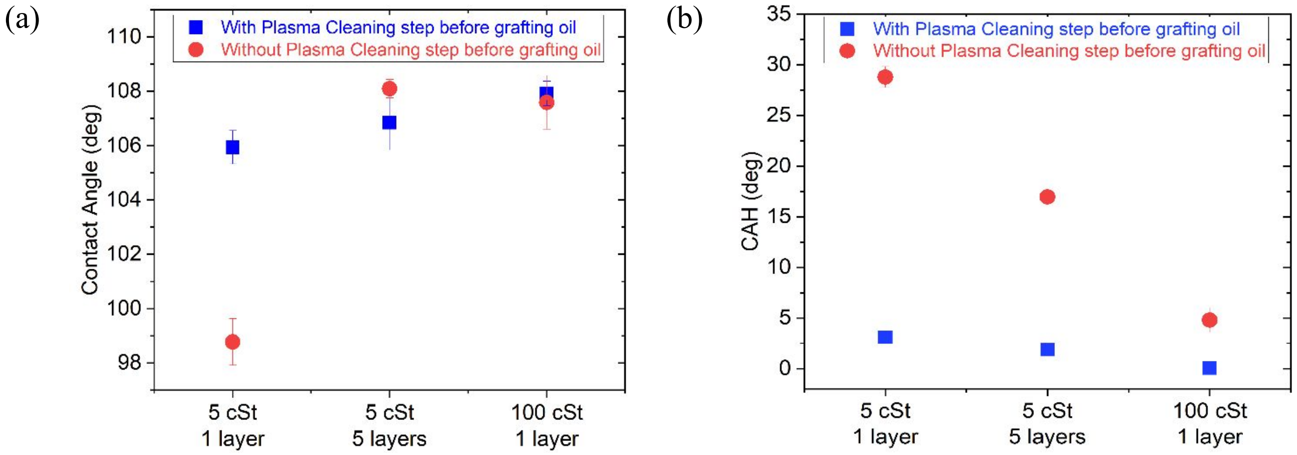

Figure SI-1: Variation in (a) CA (b) CAH for low and high viscosity oil grafted samples when prepared without plasma cleaning step (3 µL water droplet).

SI-2. Effect of temperature

Temperature is an important parameter for oil grafting as it controls both the oil evaporation as well as the grafting. It has been noticed that the samples deposited with 100 cSt viscosity oil and grafted at 300 ^oC showed preferential evaporation of oil and the surface was very rough visibly after complete evaporation of the oil. Hence, samples were grafted at 250 ^oC, which also showed uneven features. One of the cause of this non-homogenous surface grafting seemed to be the oil volume deposited onto it. So, to accommodate these two parameters, the samples were coated with 100 cSt viscosity oil via dip-coating method which deposited approximately 0.185 µL oil for one layer [1] and they were heated at 200 ^oC which left behind a visible homogenous surface. Lower viscosity oil (5 cSt) samples were also prepared at 225 ^oC and 250 ^oC. There is no clear trend observed in terms of Eq. CA, SA and CAH when temperature was changed from 225^oC to 250^oC (Figure SI-2), which is also discussed by Eifert et al. [2].

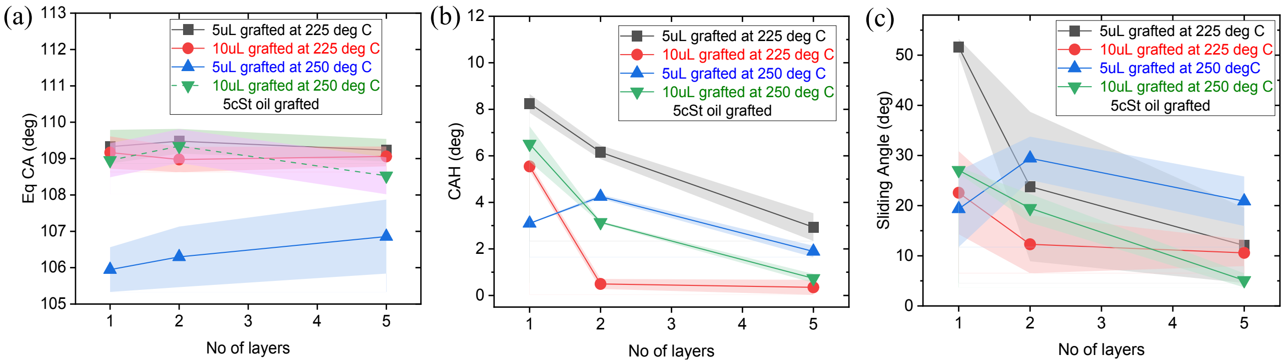

Figure SI-2: Variation in (a) Eq. CA (b) CAH (c) SA with number of layers on samples with 5 cSt oil grafted at two different temperatures (3 µL water droplet).

SI-3. Wettability analysis on copper substrate

Besides silicon as the base substrate, we prepared some samples using polished copper as substrates. We grafted 5 cSt oil (up to 5 layers) and 100 cSt oil (up to 3 layers) on the substrate following the same steps as describe in the main manuscript for silicon substrate. The results in terms of apparent contact angle and contact angle hysteresis are given below in **Error! Reference source not found.**. It can be observed that the 5 cSt oil grafted samples showed similar apparent contact angles when compared to silicon substrate, while the contact angle hysteresis increased considerably by one order of magnitude. Whereas, in case of 100 cSt oil grafted copper substrates showed only 1° or 2° increase in contact angle hysteresis. On these substrates, presumably lower wetting and more roughness of the copper substrates (as compare to atomically smooth silicon wafer) would slow down or hinder the motion of the silicone oil contact line during grafting, which may result in less uniform and less homogeneous film deposition during grafting with the consequent droplet pinning enhancement. In addition, the different affinity of PDMS brushes attachment (especially short chain length when grafting low viscosity oil) with the base substrate also may play a role in the motion of the water droplet contact line when characterising the CAH.

Table 1: Results obtained by grafting silicone oil on copper substrate and a comparison with their counter parts prepared on silicon substrate

		**Base substrate: Copper**		**Base Substrate: Silicon**	
	Number of layers grafted	Apparent Contact Angle (°)	Contact Angle Hysteresis (°)	Apparent Contact Angle (°)	Contact Angle Hysteresis (°)
5 cSt oil grafted	1	109.0 ± 0.8°	21.0 ± 2.4°	105.9 ± 0.6°	3.1 ± 0.1°
	2	109.2 ± 0.2°	32.5 ± 1.5°	106.3 ± 0.8°	4.3 ± 0.1°
	5	108.5 ± 0.3°	17.1 ± 1.0	106.9 ± 1.0°	1.9 ± 0.3°
100 cSt oil grafted	1	109.6 ± 0.4°	3.2 ± 1.5°	107.9 ± 0.5°	0.1 ± 0.1°
	2	110.8 ± 0.5°	4.9 ± 1.6°	107.9 ± 0.3°	0.3 ± 0.2°
	3	112.2 ± 0.7°	2.4 ± 1.0°	107.0 ± 0.6°	0.5 ± 0.2°

SI-4. Stability Tests

The prepared samples are tested under different conditions in order to check the robustness and stability of the coatings. These tests included sonication in ultrasonic bath to observe their mechanical stability, sonication in ethanol using ultrasonic bath to check their robustness under harsh organic solvent conditions, soaking in ethanol for longer time period (hours) to see the effect of aging in chemical environment and subjecting to high temperature steam to predict their behaviour under high temperature humid conditions. The sketches of these setups can be found in Figure SI-3.

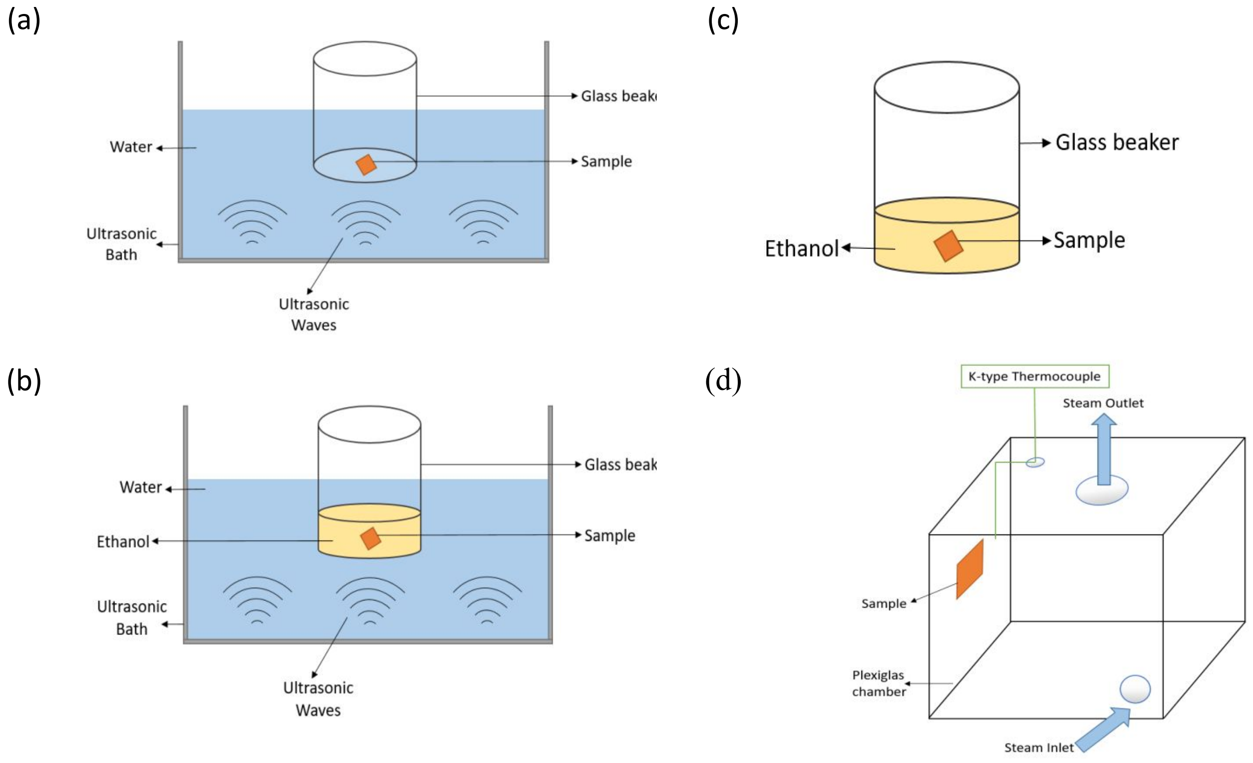

Figure SI-3: Schematic diagram of (a) Sonication setup (b) Sonication in Ethanol setup (c) Soaking in Ethanol and (d) Steam setup.

The first test is conducted to check the mechanical stability of the coatings by placing the samples in an ultrasonic bath for 30 minutes subjecting the coated surfaces to mechanical vibrations. The schematic diagram of the setup is shown in Figure SI-3a. For both 5 cSt and 100 cSt grafted oil samples, a marginal change in the apparent CA after being subjected to 1 sonication cycle in an ultrasonic bath for 30 minutes when comparing Figure SI-4a & b to Figure 7a & c in the main manuscript. In parallel, stability tests via sonication in bath of ethanol as shown in Figure SI-3b are carried out. A decrease in apparent CA is observed in the range of 3-5° for all samples independently of the number of layers and the oil viscosity as indicated in Figure SI-4 when compared to Figure 7 in the main manuscript.

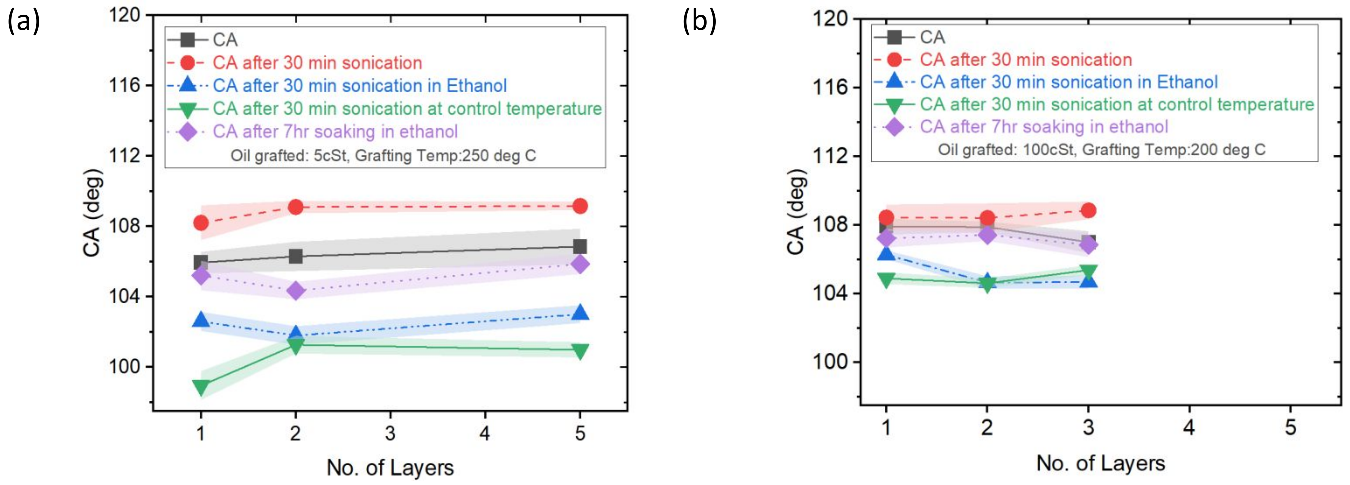

Figure SI-4: CA variation of 3 µL water droplet under various test for a) 5 cSt oil grafted samples b) 100 cSt oil grafted samples. The scale bar is 0.5 mm. Note that the CAH line (black) is overlapping with CAH after 7 hr soaking line (purple) for in figure b.

As the sonication proceeds, the temperature of both the samples as well as those of the fluids inside the ultrasonic bath increase to values near 50 ^{o}C due to the imposed vibrations. Hence, to be able to inspect whether the reported change in apparent CA is due to ultrasonic vibrations or owed to the temperature increase during the sonication process; the sonication process is repeated again with controlled water temperature (17-20 ^{o}C). Under temperature controlled conditions the greatest change in the apparent CA with a decrease down to approximately 100° is reported. A further stability test is carried out by soaking the samples in ethanol at room temperature for 7 hours as sketched in Figure SI-3c. A very small change in apparent CA is observed (Figure SI-4) proving that the change in apparent CA is owed mainly to the coupled mechanisms of sonication and temperature change, hence indicating good strength of coatings under organic solvent chemical environment.

When looking into the CAH, for 5 cSt oil grafted samples, the CAH decreased from its initial value under sonication, i.e., mechanical vibration, while the CAH increased to 17° for 1 layer of coating when subjected to sonication in ethanol bath. For both 5 cSt and 100 cSt oil grafted samples, the highest CAH is observed for samples which are subjected to sonication in ethanol bath as in Figure SI-5a & b. However, 100 cSt oil grafted samples showed better stability with CAH values below 10° for all samples under all conditions, showing that these coatings are rather robust. Though independently of the test carried out, the CAH increases when compared to original fabricated sample with mechanical 30 minutes sonication and 7 hours of ethanol soaking yielding CAH approximately 1°.

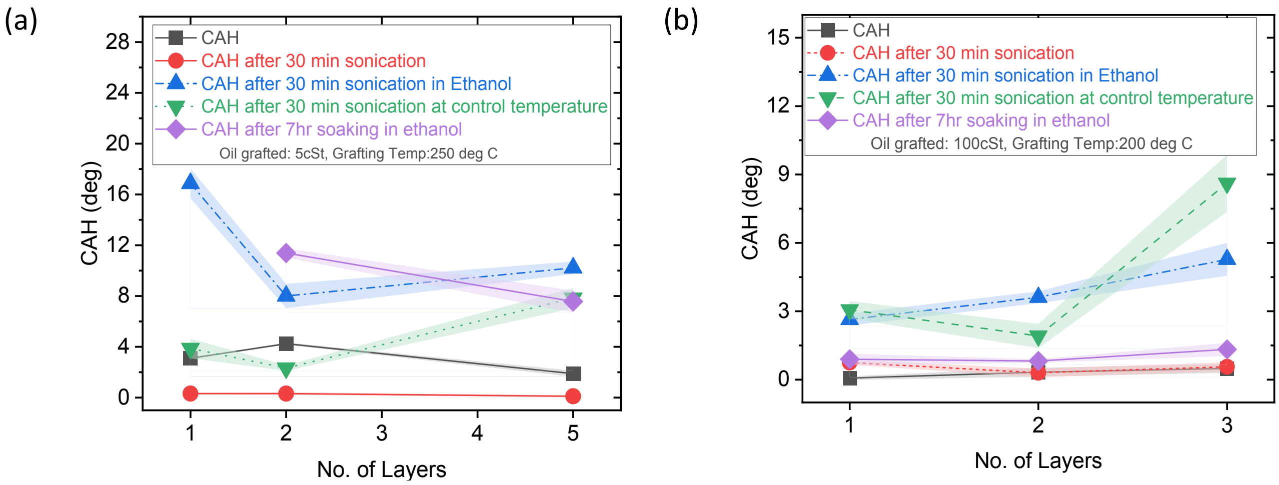

Figure SI-5: Contact Angle Hysteresis variation under various test for a) 5 cSt oil grafted samples b) 100 cSt oil grafted samples

To further examine the stability of the coatings, additional sonication as well as sonication in a bath of ethanol cycles are carried out. For the mechanical stability under sonication, stability tests are repeated for 20 cycles with each cycle lasting for 30 minutes. On one hand, for 5 cSt oil grafted surfaces, a small increase in apparent CA is observed after one cycle which is presumably due to the temperature increase due to sonication as mentioned above. However, after 2 cycles a small decrease in the apparent CA is observed which is comparable to already published literature [53] where the sonication time is 20 minutes for lubricant infused surface and no visible change in apparent CA. Our samples showed similar results even without impregnation of oil. It is worth noting that the observed decrease in the apparent CA is more pronounced as the number of layers decrease from 5 to 2 to 1 layer presumably owed to the less number and density of PDMS brushes on the surface. The apparent CA on 5cSt oil grafted surfaces under mechanical vibration reached a plateau after 15 cycles (Figure SI-6a). On the other hand, a very small change in the apparent CA is observed for 100 cSt oil grafted samples even after 20 cycles (Figure SI-6b) and all apparent CA values reported for the different sonication cycles and for the different number of layers are within their standard deviations, hence indicating a very stable robust coating.

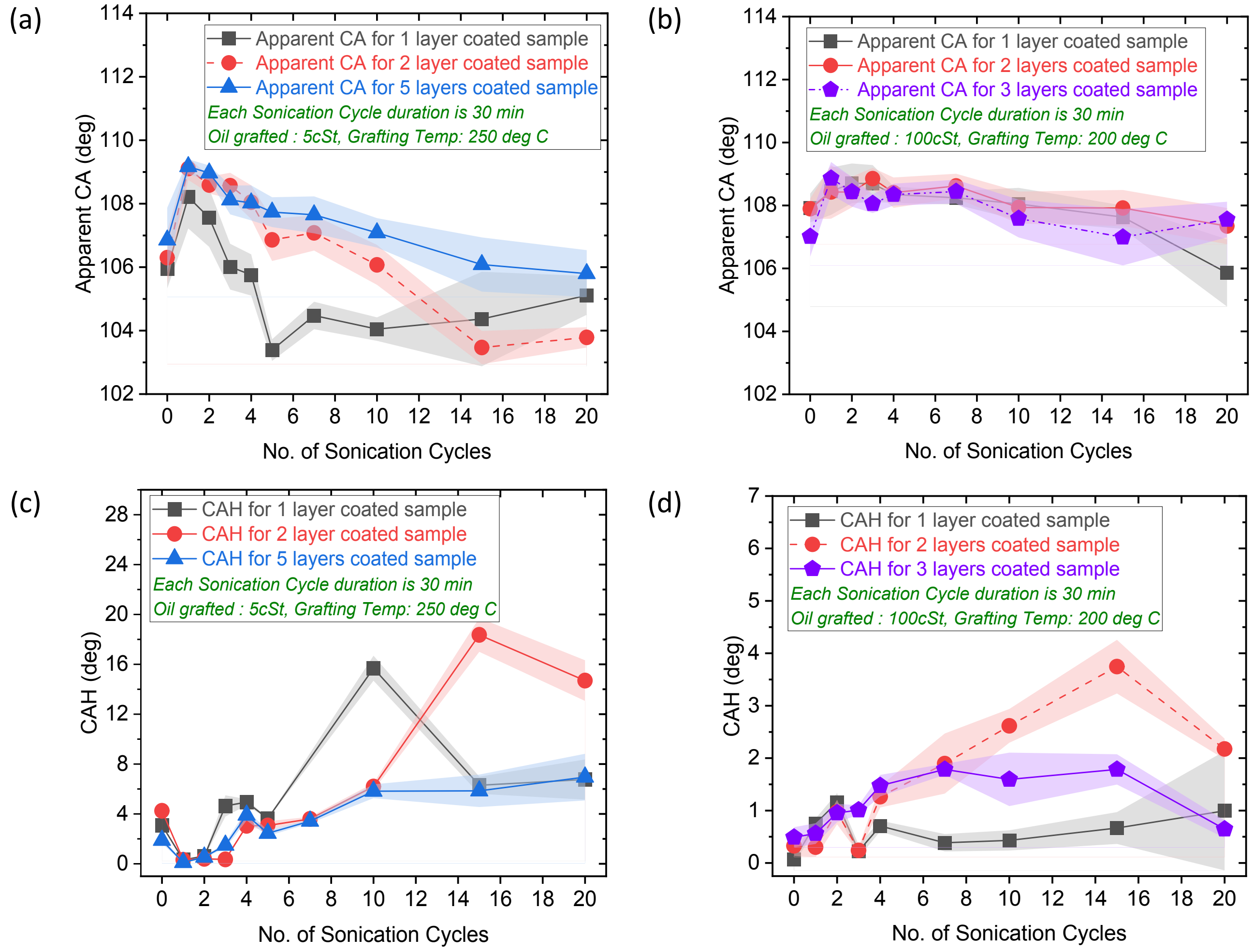

Figure SI-6: Apparent CA variation with number of Sonication Cycles a) 5 cSt oil grafted samples b) 100 cSt oil grafted samples. And Contact Angle Hysteresis variation with number of Sonication Cycles c) 5 cSt oil grafted samples d) 100 cSt oil grafted samples.

Although the apparent CA is an important metric, CAH is a better one for the determination of the adhesion. A decrease and thereafter an increase in the CAH is observed within the first 5 sonication cycles for 5 cSt (Figure SI-6c). Thereafter an increase in CAH is followed, which in the case of 5 layers is a slight increase, in the case of 2 layers a sudden increase, and in the case of 1 layer a sudden increase and then a decrease with a final CAH after 20 cycles equal to that of 5 layers and equals 8°. When looking into 100 cSt oil grafted surfaces, it is evident form Figure SI-7d that one layer coated sample is the most stable with roughly no change in CAH even after 20 sonication cycles maintaining this within 1° ±1°, i.e., no degradation on the adhesion performance. For subsequent layers, an increase in the CAH after the 3rd sonication test ensued with a maximum of 4° CAH after 15 cycles for 2 layers and up to 2 ^{o} after the 7 cycles for 5 layers.

For sonication in a bath of ethanol, the apparent CA decreased rapidly after one cycle all samples but reached a plateau after first cycle (Figure SI-7a&b). After 10 cycles, the overall decrease in the apparent CA is approximately 4° to 7° for 5 cSt oil grafted samples and 3° to 4° for 100 cSt oil grafted samples,

all with values above 100º in the hydrophobic regime. When looking into the CAH, an increase in the CAH is observed for all cases after the first and second cycles with increases as high as 14º in the case of 5 cSt and 1 layer to as low as 3º for 100 cSt and 1 layer. In all cases, samples seem to reach a plateau after 5 cycles (Figure SI-7 c&d). After 10 cycles an average CAH of 10º is reported for 5cSt 1 and 5 layers, while an average CAH of 6º can be established for 100 cSt independently of the number of layers. To note is the high increase in the CAH for 5 cSt oil grafted sample with 2 layers (Figure SI-7c).

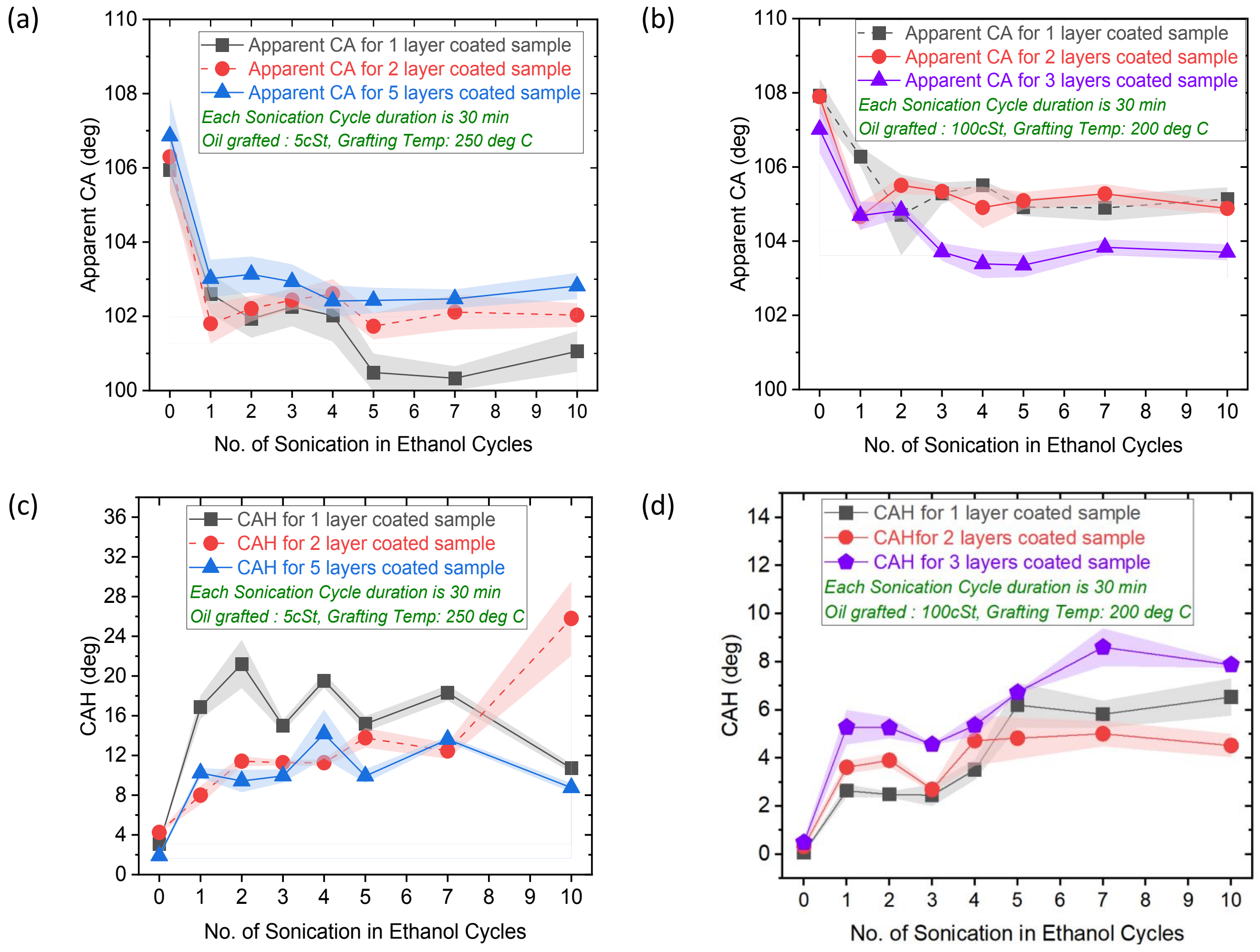

Figure SI-7: Apparent CA variation with number of Sonication in Ethanol Cycles a) 5 cSt oil grafted samples b) 100 cSt oil grafted samples. And Contact Angle Hysteresis variation with number of Sonication in Ethanol Cycles c) 5 cSt oil grafted samples d) 100 cSt oil grafted samples

To further investigate the robustness of the PDMS brushes grafted under humid and high temperature environment, samples are subjected to high temperature saturated steam (99 ± 1 ºC) for different number of cycles with a duration per cycle of 5 minutes. At first, the samples are placed directly in front of the nozzle (steam outlet with temperature 69 ± 1ºC), which, due to high pressure steam injection, resulted in PDMS brushes to shift towards the sides rendering the surface at the centre rather hydrophilic as a water droplet spreads in the centre. To minimize this effect, a Plexiglas chamber is built to contain the saturated steam with samples attached to one side of the chamber via double sided tape.

The schematic for the steam setup is shown in Figure SI-3d. By not subjecting the sample directly to the steam jet exiting the nozzle, the changes observed on the sample surface are uniform throughout. For lower viscosity oil, *i.e.*, 5 cSt, the apparent CA decreased marginally after 1 cycle but the considerable decrease is observed in 1 layer grafted sample after 3 cycles with apparent CA below 100º as in Figure SI-8a. Whereas, for all high viscosity oil grafted samples, *i.e.,* 100 cSt, independently of the number of coated layers from 1 to 3, , overall a 3 º to 4º decrease in the apparent CA is observed after the first cycle and no further change is reported even after 7 cycles of steam exposure with all apparent CA above 104º (Figure SI-8b).

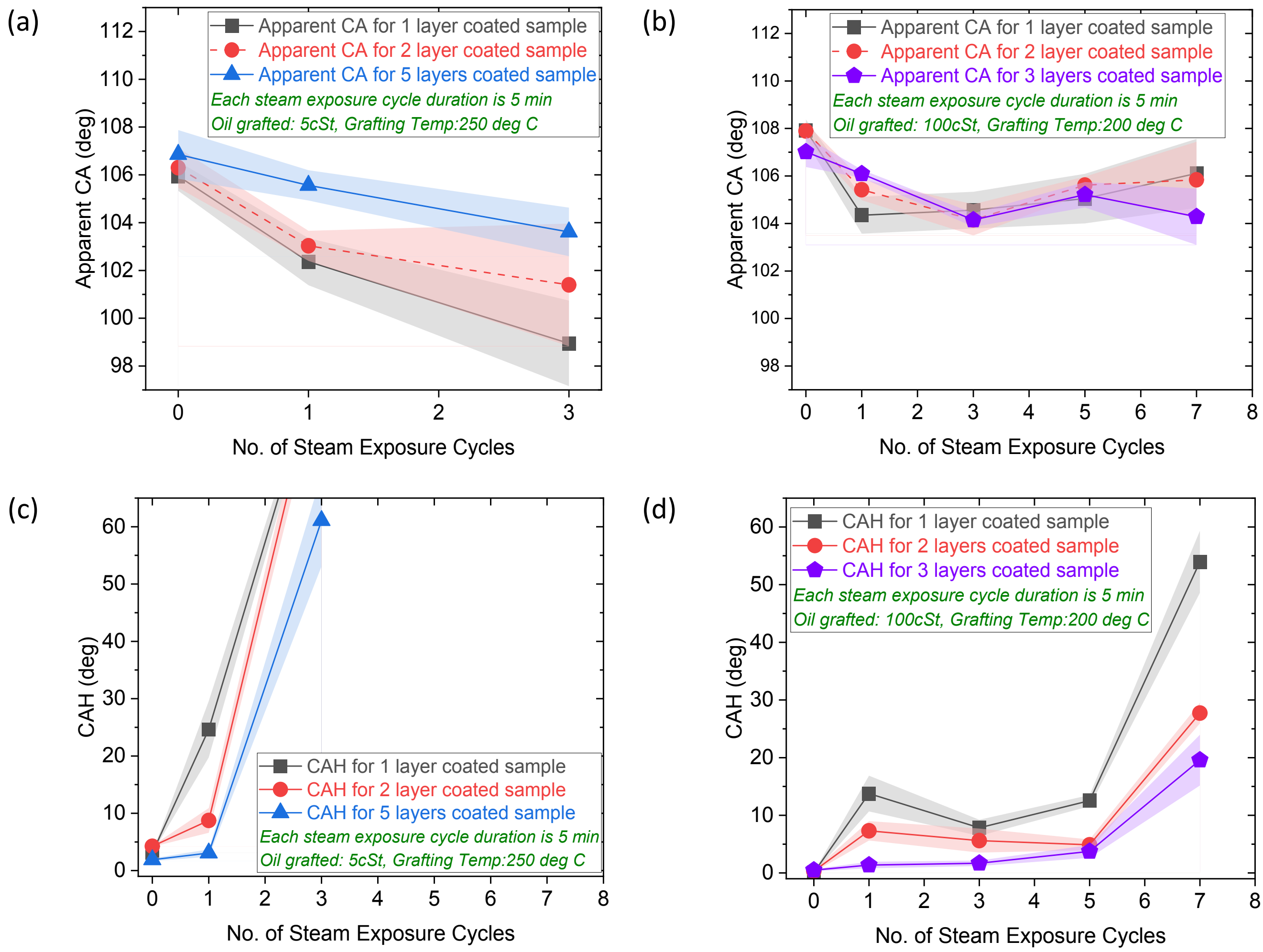

Figure SI-8: Apparent CA variation with number of Steam Cycles a) 5 cSt oil grafted samples b) 100 cSt oil grafted samples. And Contact Angle Hysteresis variation with number of Steam Cycles c) 5 cSt oil grafted samples d) 100 cSt oil grafted samples.

In terms of CAH, for 5 cSt oil grafted samples, a substantial increase is perceived for 1 layer coated samples after 1 cycle while 2 and 5 layers coated samples showed CAH below 10º. When these samples are subjected to additional 2 cycles, 1 layer and 2 layer coated samples showed contact line pinning

and 5 layers coated sample also exhibited high CAH (roughly around 60°) (Figure SI-8c). On the other hand, for high viscosity oil grafted samples, the increase is CAH is very small below 10° for 2 and 3 layer grafted samples until 5 cycles of steam exposure. Thereafter, the CAH increases sharply after that with CAH equal or above 20 ° as represented in Figure SI-8d. 1 layer and 3 layers grafted sample showed highest and lowest hysteresis respectively at each point. The 100 cSt oil grafted samples showed very good results in terms of wettability (high apparent CA) adhesion (low SA as well as low CAH) and stability with low change of apparent CA, CAH and SA throughout the different samples.

References:

[1] S. J. Goodband, S. Armstrong, H. Kusumaatmaja, and K. Voïtchovsky, Effect of Ageing on the Structure and Properties of Model Liquid-Infused Surfaces *Langmuir* 2020, 36 (13), 3461–3470.

[2] A. Eifert, D. Paulssen, S. N. Varanakkottu, T. Baier, and S. Hardt, Simple Fabrication of Robust Water-Repellent Surfaces with Low Contact-Angle Hysteresis Based on Impregnation, *Advanced Materials Interfaces* 2014, 1 (3), 1–5.
